# Selective Synthesis of *N*-Acylnortropane Derivatives in Palladium-Catalysed Aminocarbonylation

**DOI:** 10.3390/molecules26061813

**Published:** 2021-03-23

**Authors:** László Kollár, Ádám Erdélyi, Haroon Rasheed, Attila Takács

**Affiliations:** 1 Department of Inorganic Chemistry, Faculty of Sciences, University of Pécs, Ifjúság útja 6, H-7624 Pécs, Hungary; kollar@gamma.ttk.pte.hu (L.K.); erdelyi.adam99@gmail.com (Á.E.); privateharoon@gmail.com (H.R.); 2 János Szentágothai Research Centre, University of Pécs, Ifjúság útja 20, H-7624 Pécs, Hungary; 3 MTA-PTE Research Group for Selective Chemical Syntheses, Ifjúság útja 6, H-7624 Pécs, Hungary

**Keywords:** aminocarbonylation, *N*-acylnortropanes, carbon monoxide, palladium

## Abstract

The aminocarbonylation of various alkenyl and (hetero)aryl iodides was carried out using tropane-based amines of biological importance, such as 8-azabicyclo[3.2.1]octan-3-one (nortropinone) and 3α-hydroxy-8-azabicyclo[3.2.1]octane (nortropine) as *N*-nucleophile. Using iodoalkenes, the two nucleophiles were selectively converted to the corresponding amide in the presence of Pd(OAc)_2_/2 PPh_3_ catalysts. In the presence of several iodo(hetero)arenes, the application of the bidentate Xantphos was necessary to produce the target compounds selectively. The new carboxamides of varied structure, formed in palladium-catalyzed aminocarbonylation reactions, were isolated and fully characterized. In this way, a novel synthetic method has been developed for the producing of *N*-acylnortropane derivatives of biological importance.

## 1. Introduction

Amide functionality can be found in numerous pharmaceutically, biologically active and agrochemical molecules [1,2,3], and its formation has been deeply studied [4,5]. There are several ways of introducing acyl functionality into simple model compounds or skeletons of practical importance. The application of conventional acylating agents is well known and is referenced in reviews [6], handbooks, and even textbooks [7]. Activated carboxy compounds are widely used acylating agents producing the desired amide derivative in the presence of amines. Acyl azides [8], anhydrides [9,10,11], as well as esters [12,13] can be successfully used in the formation of amide bond. The most efficient and widely-used method among the conventional amide synthesis is the reaction with acyl chlorides and amines [14,15,16,17,18]. Despite of the widespread use of acyl chlorides in amide synthesis, it has to be noted that acyl chlorides have significant limitations (e.g., hydrolysis, racemization, cleavage of protecting groups) [6]. Due to the importance of amides, several novel methods have also been developed for their synthesis [19,20,21,22].

The palladium-catalyzed aminocarbonylation is also an effective and widespread synthetic method for producing amides. Since the discovery of the aminocarbonylation by Heck et al. (“Heck-carbonylation”) [23,24,25], the use of a primary or secondary amine, carbon monoxide, and aryl/alkenyl halides (or their synthetic surrogates, aryl triflates, and alkenyl triflates) in the presence of palladium catalysts leads to carboxamides in great variety [26,27,28,29,30,31,32,33,34,35,36,37]. Several α,β-unsaturated carboxamides and aryl carboxamides, using palladium-catalyzed aminocarbonylation as a key reaction, were recently synthesized in our laboratory [38,39,40,41,42,43,44,45]. Just to illustrate the most recent developments, the functionalization of aromatic *N*-heterocycles in aminocarbonylation should be mentioned [46,47,48,49].

When the hundreds of examples of aminocarbonylation reactions are surveyed, we might conclude that great variety of aryl/alkenyl halides/triflates were investigated as substrates in the presence of simple primary or as secondary amines as *N*-nucleophiles. However, a relatively small number of investigations are focused on the “opposite” reaction, i.e., applying amines of practical (for instance, pharmaceutical) importance and palladium-acyl intermediates formed in situ from relatively simple aryl or alkenyl halide by oxidative addition followed by carbon monoxide insertion. The palladium(II)-acyl species can be considered as highly reactive acylating agents [50,51,52].

Considering the fact that the palladium-catalyzed aminocarbonylation reactions have been widely applied to build-up amide functionality in several bioactive compounds and natural products, this method has been chosen to produce novel *N*-acylnortropane derivatives in our research. The high selectivity, the mild reaction conditions, and the tolerance of wide range of functional groups make also more applicable the palladium-catalyzed aminocarbonylation for the synthesis of our target compounds than the conventional acylating methods.

The tropane-based derivatives have great importance in chemistry, which have been indicated by their wide-ranging use. The synthesis of *N*-substituted nortropinones via reactivity umpolung of tropinone has been carried out by Willand et al. [53]. The role of twist-boat conformers in hydride reduction of tropinone has been investigated computationally and compared with experiments [54]. The diastereoselective acetylation of 6,7-dihydroxytropinones has been investigated in enzyme-catalyzed reaction [55]. Recently, a tropine-based ionic liquid was used for the resolution of racemic amino acids [56].

The functionalization of the tropane skeleton can be carried out efficiently in homogeneous catalytic reactions. The nonaflation–Heck reaction protocol was used for the synthesis of 1,3-tropadiene [57]. The palladium-catalyzed alkoxycarbonylation of the enol-triflate obtained from tropinone afforded the three epimers of diester in the presence of ethylene glycol [58]. The vinylboronate derivative obtained from tropinone underwent Suzuki–Miyaura coupling with aryl bromides resulting in aryl substituted nortopanes [59]. 3-Iodo-2-tropene was transformed to the corresponding 3-carboxamides and 3-esters in palladium-catalyzed amino- and alkoxycarbonylation, respectively [60]. A tropane-ring heterocyclic compound, scopolamine was synthesized in rhodium-catalyzed [4 + 3] cycloaddition reaction [61].

To the best of our knowledge, only just a few aromatic or α,β-unsaturated *N*-acyl nortropane derivatives can be found in the literature [62,63], and the using of nortropinone and nortropine as nucleophiles in aminocarbonylation has not been mentioned. It has to also be noted that the tropane-based derivatives have enormous biological relevance [64]. As a part of our long-standing interest in the investigation of the fine details of aminocarbonylation, we decided to investigate of use of tropane-derived nucleophiles in aminocarbonylation.

In this paper, the selective synthesis of *N*-acylnortropane derivatives, possessing a variety of *N*-acyl moieties will be described.

## 2. Results

### 2.1. Aminocarbonylation of Iodoalkenes (***1***–***5***) in the Presence of Tropane-Based Nucleophiles (***a***, ***b***)

**Scheme 1:**
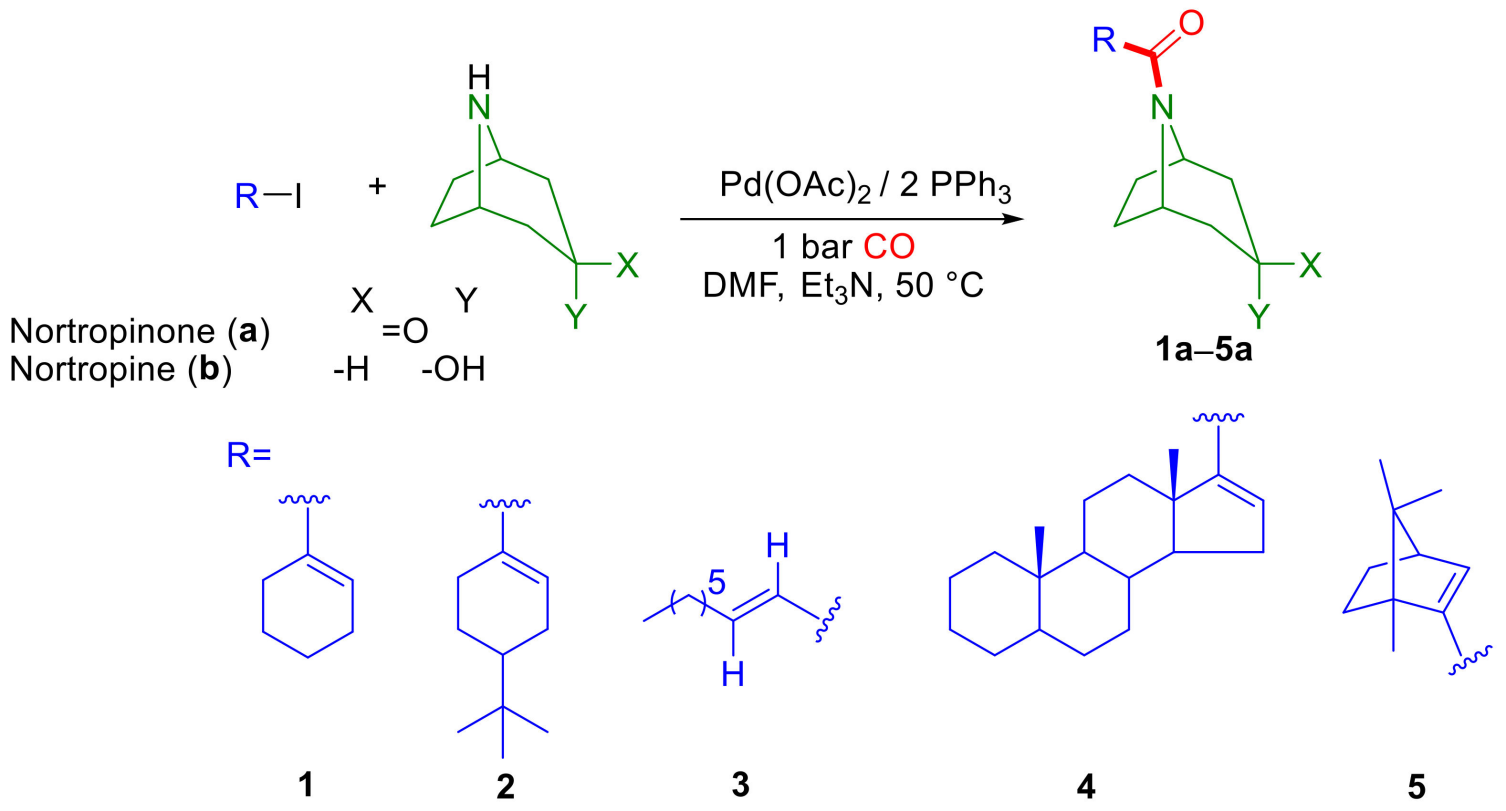
Aminocarbonylation of tropane-based nucleophiles (**a**, **b**) in the presence of iodoalkenes (**1**–**5**) using Pd(OAc)_2_ +2 PPh_3_ in situ catalysts.

Azabicyclo[3.2.1]octan-3-one (nortropinone) (**a**) and 3α-hydroxy-8-azabicyclo[3.2.1]octane (nortropine) (**b**) as *N*-nucleophiles were applied for the aminocarbonylation of iodoalkenes (such as 1-iodocyclohexene (**1**), 4-*tert*-butyl-1-iodocyclohexene (**2**), *trans*-1-iodo-1-octene (**3**), 17-iodoandrost-16-ene (**4**), and 2-iodobornene (**5**)) in the presence of palladium catalysts (Scheme 1). Based on the prior knowledge of our research group [65,66], a low-ligated, highly active Pd(0) catalyst, prepared in situ from Pd(OAc)_2_ and PPh_3_, was used. The reduction of Pd(II) to catalytically active, highly unsaturated Pd(0) species took place on the influence of the phosphine while it is oxidized to phosphine oxide [67,68,69].

The investigation of aminocarbonylation of nortropane-based nucleophiles was started with iodoalkenes (**1**–**5**), as shown on Scheme 1 and in Table 1. Nortropinone (**a**) was reacted with 1-iodocyclohexene (**1**) under atmospheric CO pressure. The target compound was observed just in traces by GC (entry 1) in short reaction time. Surprisingly, just 42% conversion was observed after 24 h (entry 2), and the complete conversion was not reached still after 48 h stirring (entry 3). Using elevated carbon monoxide pressure (40 bar), the substrate **1** was completely transformed to the corresponding carboxamide (**1a**) after 24 h (entry 5). It has to be noted, that double carbon monoxide insertion was not observed under these conditions. It can be stated, based on this experiment and our previous results, that the aminocarbonylation reactions of iodoalkenes do not provide the 2-keto-carboxamide type products [70,71]. In the presence of substrates **2** and **3**, shorter reaction time (entries 6 and 7) was necessary to reach the complete conversion than in the case of **4** and **5** (entries 8 and 10). The *N*-acyl-nortropinone derivatives (**1a**–**5a**), synthesized in the aminocarbonylation reactions, were isolated in good yields (77–86%).

The aminocarbonylation of nortropine (**b**) was also carried out in the presence of the above mentioned substrates (**1**–**5**). While the complete conversion of 17-iodoandrost-16-ene (**4**) to the corresponding carboxamide (**4b**) required 6 h (entry 15), the other substrates (**1**–**3**, **5**) were fully converted in 1 h (entries 11–13, 16).

Considering the behavior of the two nortropane-based nucleophiles, used in the reactions, decreased reactivity was observed in the case of nortropinone (**a**): the starting substrates (**1**–**5**) were completely converted into the target carboxamides in substantially longer reaction times (4–24 h). This phenomenon can be explained by the different conformation of the two nucleophiles. We believe that the different reactivity of the two secondary amines is due to steric factors. That is, the close to perfect chair conformation of nortropine is highly distorted when an sp^2^ carbon (that is, keto functionality) is introduced into the ring. This distortion could result in different reactivities both in the amine activation step toward the formation of the Pd-amide intermediate, and also in the product-forming step, where the acyl–amide–palladium(II) complex undergoes reductive elimination.

### 2.2. Aminocarbonylation of Iodoarenes (***6***–***10***) in the Presence of Tropane-Based Nucleophiles (***a***, ***b***)

After completing the reactions with the above mentioned iodoalkene substrates, we turned our attention to the investigation of aminocarbonylation of iodoarene compounds in the presence of nortropinone (**a**) and nortropine (**b**).

#### 2.2.1. Optimization Study with Iodobenzene (**6**)

**Scheme 2:**
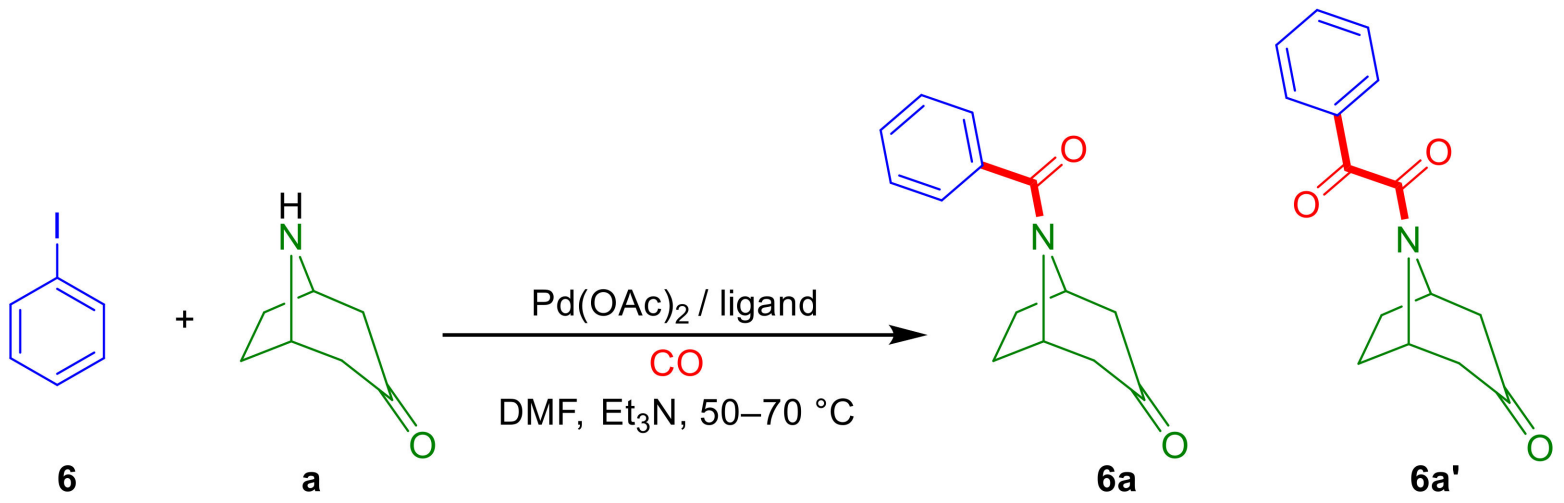
Aminocarbonylation of iodobenzene (**6**) in the presence of nortropinone (**a**).

The aminocarbonylation of iodobenzene (**6**) was carried out in the presence of nortropinone (**a**) and nortropine (**b**) under different reaction conditions to find to optimal parameters for the further investigations (Scheme 2). Pd(OAc)_2_ was used as the catalyst precursor, and the influence of ligand, CO pressure, and temperature on the reactivity and selectivity was investigated.

**Figure 1:**
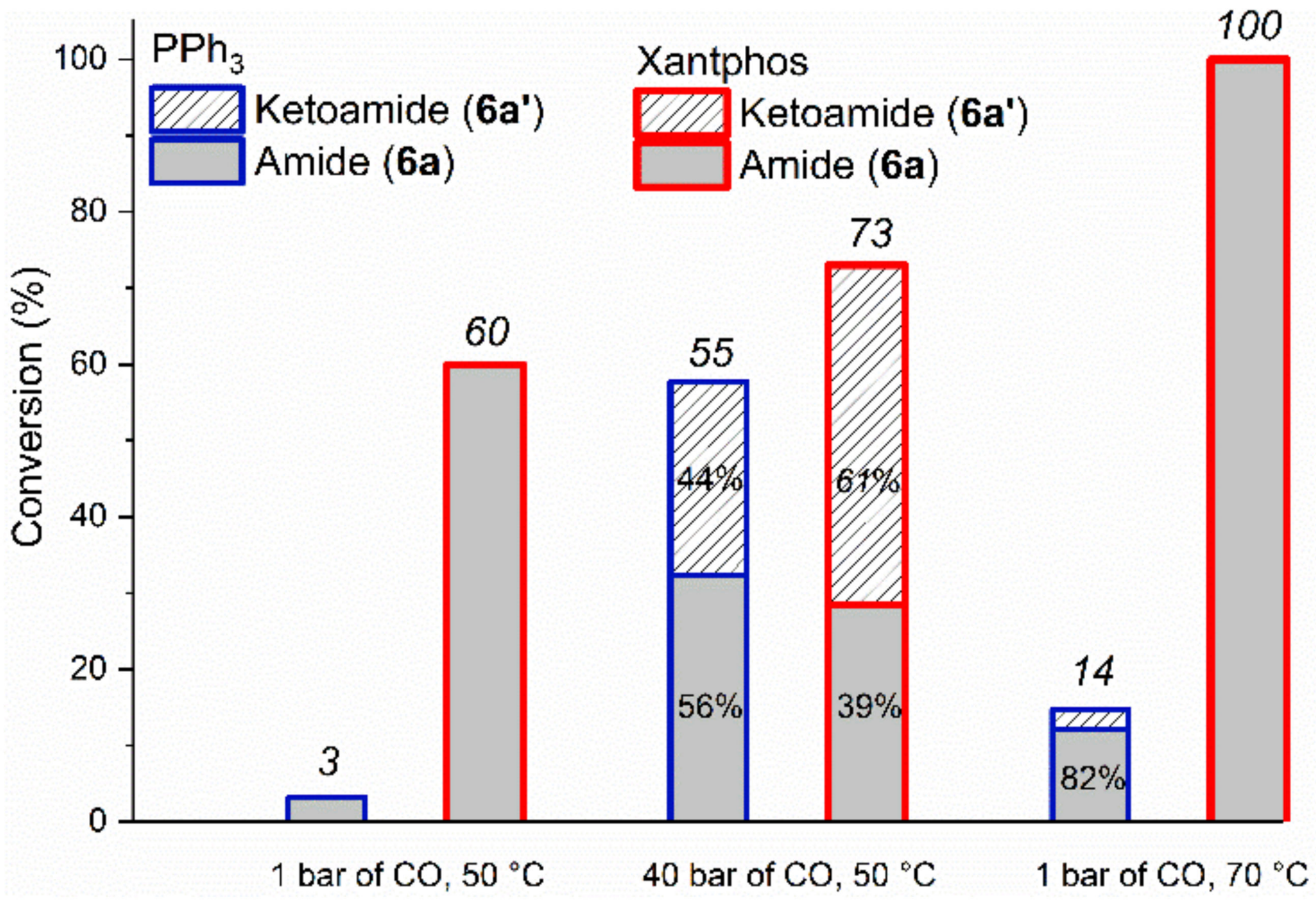
Conversion of **6** in the presence of **a** as a function of reaction conditions used during their aminocarbonylation in DMF after 24 h.

Performing the reactions in the presence of PPh_3_ at 1 bar of CO, it can be seen, that the conversion was very low both at 50 and 70 °C (Figure 1). Using elevated carbon monoxide pressure (40 bar), 55% conversion was detected after 24 h. Under high pressure conditions, the corresponding 2-keto-carboxamide (**6a’**) was also formed due to the second carbon monoxide insertion. The ratio of amide (**6a**): ketoamide (**6a’**) type products was 56:44.

Based on our previous results [72], the PPh_3_ was changed to the bidentate Xantphos to reach complete conversion in sort reaction time. Surprisingly, under atmospheric conditions, only 60 % conversion was observed after 24 h in a selective reaction towards the corresponding carboxamide (**6a**). Higher conversion (73%) was detected under elevated CO pressure (40 bar) and the ratio of **6a**:**6a’** was 39:61. Increasing the temperature to 70 °C, the target carboxamide (**6a**) was synthesized selectively after 6 h under atmospheric conditions (Figure 1).

**Scheme 3:**
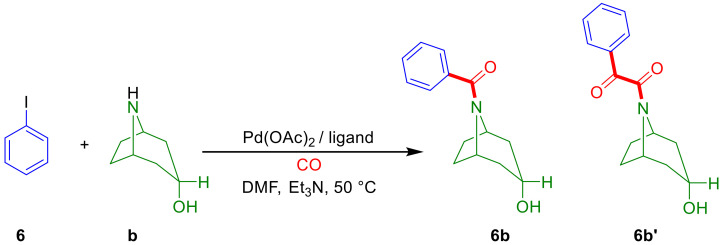
Aminocarbonylation of iodobenzene (**6**) in the presence of nortropine (**b**).

The optimization study was also performed in the reaction on iodobenzene (**6**) with nortropine (**b**) under aminocarbonylation conditions (Scheme 3).

**Figure 2:**
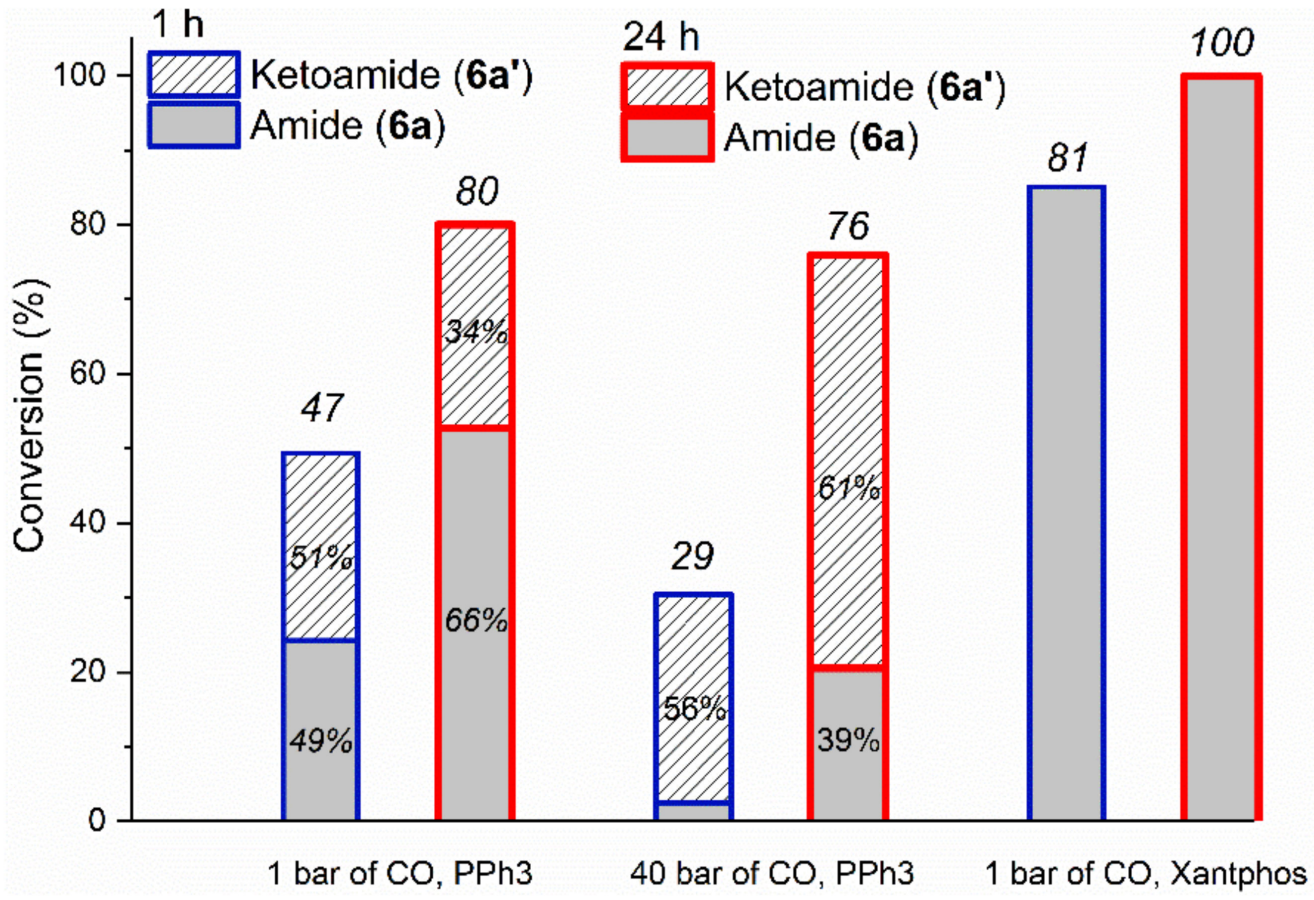
Conversion of **6** in the presence of **b** as a function of carbon monoxide pressure and the ligand used during the aminocarbonylation in DMF in 24 h stirring.

Performing the reactions in the presence of PPh_3_ both under atmospheric and 40 bar CO pressure, after 24 h, the conversion was just 80% and 76%, respectively. After 24 h stirring, the ratio of the amide (**6b**):ketoamide (**6b’**) type products was 66:34 under atmospheric CO pressure, while the chemoselectivity was shifted toward **6b’** at 40 bar (73%). Changing the PPh_3_ to the bidentate Xantphos, as we expected, the iodobenzene was completely converted after 2 h at 50 °C, producing the target carboxamide (**6b**) selectively (Figure 2). It has to be also mentioned, comparing the reactions of both nortropine-based nucleophile, that the nortropinone (**b**) showed higher reactivity than the nortropinone under same reaction conditions.

#### 2.2.2. Extending the Scope of Iodoarene Substrates

**Scheme 4:**
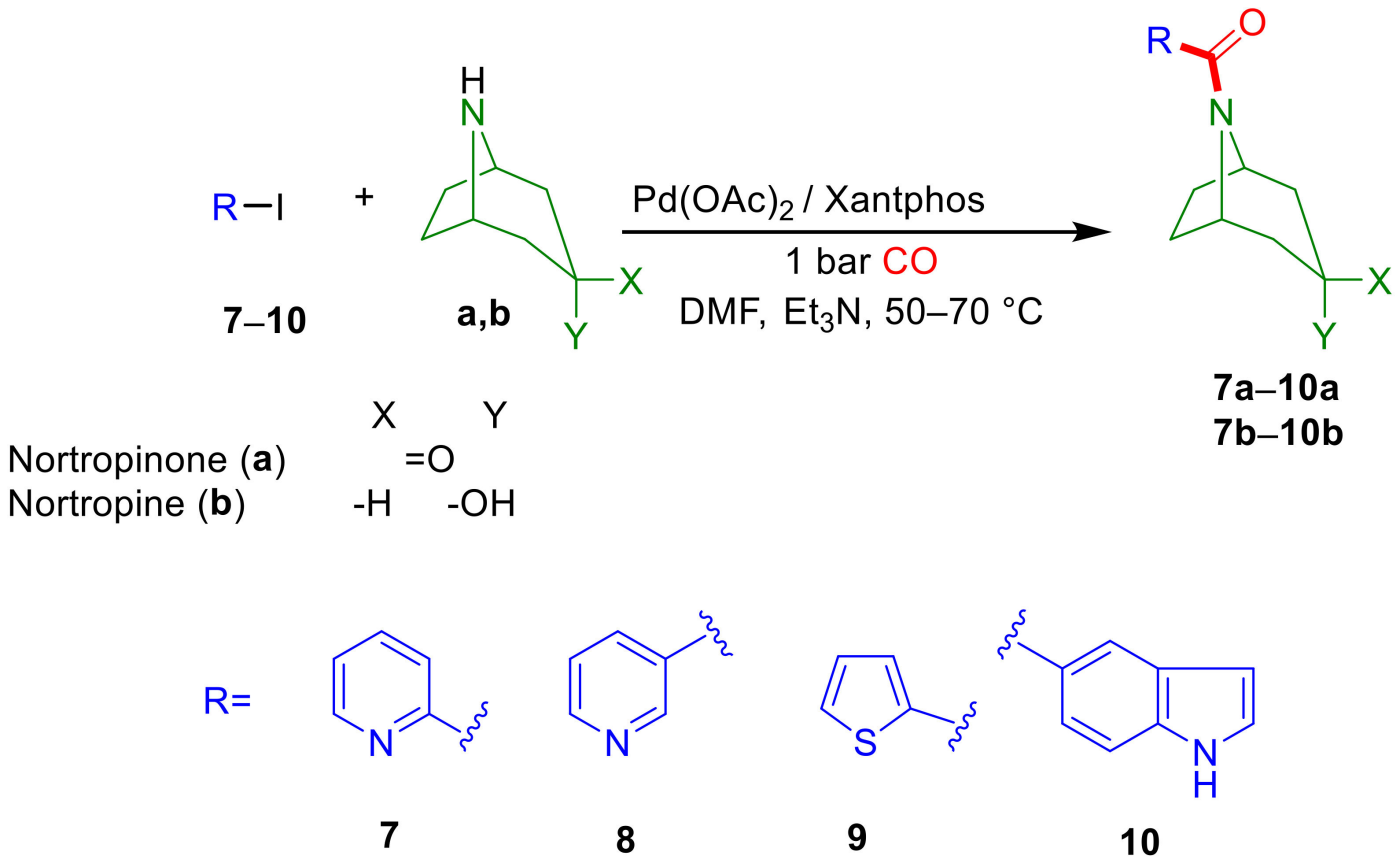
Aminocarbonylation of iodo-heteroaromatic substrates (**7**–**10**) in the presence of nortropane-based nucleophiles (**a**, **b**).

According to the results of the paragraph in Section 2.1, it can be seen that the Xantphos could be a good choice to produce selectively *N*-acyl-nortropane derivatives selectively under mild aminocarbonylation conditions in the presence of other iodo-heteroaromatic substrates. In this way, 2-iodopyridine (**7**), 3-iodopyridine (**8**), 2-iodothiophene (**9**), and 5-iodoindole (**10**) were reacted with nortropinone (**a**) and nortropine (**b**) under aminocarbonylation conditions in the presence of Pd(OAc)_2_/Xantphos catalysts (Scheme 4, Table 2).

Nitrogen-containing iodo-heteroarene substrates (**7**, **8**) were converted completely into the correspondig carboxamides (**7a**, **8a**, **7b**, **8b**) after 2–6 h reaction time (entries 1, 2, 9, 10). The difference between the reactivity of nortropinone (**a**) and nortropine (**b**) can easily be seen in the reactions with 2-iodothiophene (**9**) and 5-iodoinole (**10**). While in the presence of **b**, the reaction was complete with substrates **9** and **10** in short reaction time (entries 12, 14); using nucleophile **a**, the reaction was conspicuously slower (24–48 h) (entries 5, 7, 8). The *N*-acylnortropane derivatives synthesized in the reactions with substrates **7**–**10** were isolated in good to high yield (except **10b**).

## 3. Materials and Methods

### 3.1. General Procedures

^1^H and ^13^C NMR spectra were recorded in CDCl_3_ or DMSO-*d*_6_ on a Bruker Avance III 500 spectrometer (Bruker BioSpin Corp., Karlsruhe, Germany) at 500 and 125.7 MHz, respectively (See Supplementary Materials). Chemical shifts δ are reported in ppm relative to CDCl_3_ (7.26 and 77.00 ppm for ^1^H and ^13^C, respectively) or DMSO-*d*_6_ (2.50 and 39.50 ppm for ^1^H and ^13^C, respectively). Elemental analyses were measured on a 1108 Carlo Erba apparatus (Carlo Erba Reagents SAS, Val de Reuil, France). The FT-IR spectra were taken in KBr pellets using a Nicolet IMPACT 400 spectrometer (Thermo Fisher Scientific, Waltham, MA, USA) applying a DTGS detector in the region of 400–4000 cm^−1^; the resolution was 4 cm^−1^. The amount of the samples was ca. 0.5 mg. Samples of the catalytic reactions were analysed with Chrom-Card Trace GC-Focus GC gas-chromatograph (Thermo Fisher Scientific, Waltham, MA, USA) fitted with a capillary column coated with OV-1 (injector temp. 250 °C; oven: starting temp. 50 °C (hold-time 1 min), heating rate 15 °C min^−1^, final temp. 320 °C (hold-time 11 min); detector temp. 280 °C; carrier gas: helium (rate: 1 mL min^−1^)). Mass spectrometry data have been obtained using a GC–MS system consisting of a Perkin Elmer AutoSystem XL gas-chromatograph (PerkinElmer Life and Analytical Sciences, Shelton, CT, USA). Melting points were measured with a Büchi apparatus (Büchi Labortechnik AG, Flawil, Switzerland). TLC plates (silica gel on TLC Al foils with fluorescence indicator 254 nm) were purchased from Sigma-Aldrich (St. Louis, MO, USA). The eluents used in thin-layer chromatography are specified below.

The Pd(OAc)_2_, the ligands (PPh_3_ and Xantphos), and the solvents were used without further purification. *trans*-1-Iodo-1-octene (**3**) and iodo-(hetero)aromatic substrates (**6**–**10**) were purchased from Sigma-Aldrich and were used without any further purification. The amine nucleophiles (nortropinone (**a**) and nortropine (**b**)) were purchased from TCI Chemicals and were used without further purification. 1-Iodocyclohexene (**1**) [73], 4-*tert*-butyl-1-iodocyclohexene (**2**) [73], 17-iodoandrost-16-ene (**4**) [74], and 2-iodobornene (**5**) were synthesized by the modified Barton-procedure [75,76]. The compound **6a** is known, and the characterization data were in accordance with the literature. The **6b** compound is also described, but its melting point is known only. ^1^H and ^13^C NMR data for these compounds are provided here for the sake of completeness.

### 3.2. Aminocarbonylation of Nortropane Derivatives (***a***, ***b***) in the Presence of Iodoalkenes (***1***–***5***) and Iodoarenes (***6***–***10***) under Atmospheric Carbon Monoxide Pressure

In a typical experiment Pd(OAc)_2_ (5.6 mg, 0.025 mmol), triphenylphosphine (13.1 mg, 0.05 mmol) or Xantphos (14.5 mg, 0.025 mmol), iodoalkene (**1**–**5**) or iodo-(hetero)arene (**6**–**11**) substrates (0.5 mmol), tropane-based nucleophiles (nortopinone (**a**), nortropine (**b**)) (0.55 mmol), and triethylamine (0.5 mL) were dissolved in DMF (10 mL) under argon in a 100 mL three-necked flask equipped with reflux condenser connected to a balloon filled with argon. The atmosphere was changed to carbon monoxide. The reaction was conducted for the given reaction time upon stirring at 50 or 70 °C and analyzed by GC and GC–MS. The cooled reaction mixture was then concentrated and evaporated to dryness under reduced pressure. The residue was dissolved in chloroform (20 mL) and washed twice with water (20 mL). The organic phase was dried over Na_2_SO_4_, filtered and evaporated under reduced pressure to a solid material. All compounds were subjected to column chromatography (Silicagel 60 (Sigma), 0.063–0.200 mm), CHCl_3_/MeOH or CHCl_3_/EtOAc eluent mixtures (the exact ratios are specified in Characterization (Section 3.4) for each compound).

### 3.3. Aminocarbonylation of Nortropane Derivatives (***a***, ***b***) in the Presence of Iodoalkenes (***1***–***5***) and Iodoarenes (***6***–***11***) under High Carbon Monoxide Pressure

In a typical experiment Pd(OAc)_2_, triphenylphosphine or Xantphos, iodoalkene (**1**–**5**) or iodo-(hetero)arene (**6**–**11**) and tropane-based nucleophile (**a**, **b**) and triethylamine were used in the same amount as above and were dissolved in 10 mL of DMF under argon in a 100 mL autoclave. The atmosphere was changed to carbon monoxide, and the autoclave was pressurized to the given pressure with carbon monoxide (caution: high pressure carbon monoxide should only be used with adequate ventilation (hood) using CO sensors as well). The reaction was conducted for the given reaction time upon stirring at 50 °C. After the given reaction time, the reaction mixture was cooled to room temperature, and the autoclave was carefully depressurized in a well-ventilated hood. The product mixture was analyzed by GC and GC–MS. The work-up of the reaction mixture was identical to that discussed for the atmospheric experiments.

### 3.4. Characterization of the Products

*(1R,5S)-8-(Cyclohex-1-enecarbonyl)-8-azabicyclo[3.2.1]octan-3-one* (**1a**), yield: 94 mg (80%); colourless oil; [Anal. Calcd. for C_14_H_19_NO_2_: C, 72.07; H, 8.21; N, 6.0. Found: C, 71.87; H, 8.16; N, 6.10]; *R*_f_ (30 % EtOAc, 70 % CHCl_3_) 0.5; ^1^H NMR (500 MHz, CDCl_3_): 5.99 (1 H, s, C=C*H*), 4.69 (2 H, br s, N-*CH*), 2.62 (2 H, br s, CH-*CH_a_*H_b_), 2.37 (2 H, d, 16 Hz, C(O)-CH_a_*H_b_*), 2.27–2.18 (2 H, m, cyclohex. *CH_2_*), 2.14–2.07 (2 H, br s, CH- CH_a_*H_b_*), 2.06–2.00 (2 H, m, cyclohex. *CH_2_*), 1.73–1.64 (4 H, m, overlapping of cyclohex. *CH_2_* and C(O)-*CH_a_*H_b_*),* 1.63–1.57 (2 H, m cyclohex. *CH_2_*); ^13^C NMR (125 MHz, CDCl_3_): 207.7, 170.2, 134.5, 129.4, 54.8 (br s, 2 C), 49.4 (br s, 2 C), 28.9 (br s, 2 C), 25.8, 24.7, 22.0, 21.6. MS m/z (rel. int.): 233 (26, [M]^+^), 205 (6), 176 (7), 148 (3), 124 (19), 109 (100), 81 (67), 53 (27). IR (KBr, ν (cm^−1^)): 1716 (CO), 1655 (C=C), 1628 (Amide I.), 1413.

*(1R,5S)-8-(4-(tert-Butyl)cyclohex-1-enecarbonyl)-8-azabicyclo[3.2.1]octan-3-one* (**2a**) (diastereomers can be distinguished), yield: 125 mg (86%); white solid, m.p. 123–124 °C, [Anal. Calcd. for C_18_H_27_NO_2_: C, 74.70; H, 9.40; N, 4.80. Found: C, 74.97; H, 9.44; N, 4.93]; R_f_ (10 % EtOAc, 90 % CHCl_3_) 0.66; ^1^H NMR (500 MHz, CDCl_3_): 7.11–7.07/6.08–6.02 (1 H, m, C=*CH*), 4.78 (2 H, br s, N-*CH*), 2.68 (2 H, br s, CH-*CH_a_*H_b_), 2.51 (1 H, m, *CH*-C(CH_3_)_3_), 2.42 (2 H, d, 16Hz, C(O)-CH_a_*H_b_*), 2.32–2.20 (2 H, m, cyclohex. *CH_2_*), 2.13–2.06 (2 H, br s, CH-CH_a_*H_b_*), 1.98–1.87 (2 H, m, cyclohex. *CH_2_*), 1.80–1.68 (2 H, m, C(O)-*CH_a_*H_b_), 1.41–1.23 (2 H, m, cyclohex. *CH_2_*), 0.89 (9 H, s, C(*CH_3_*)_3_); ^13^C NMR (125 MHz, CDCl_3_): 207.7, 170.9/170.1, 140.9/134.3, 129.9/129.8, 55.3 (br s, 2 C), 49.5 (br s, 2 C), 43.5/43.2, 32.2/32.1, 28.7 (br s, 2 C), 27.7/27.5, 27.1, 26.6/26.5, 23.6/23.5. MS m/z (rel. int.): 289 (52, [M]^+^), 274 (10), 261 (6), 232 (24), 204 (8), 165 (100), 124 (40), 81 (39), 57 (53). IR (KBr, ν (cm^−1^)): 1714 (CO), 1663 (C=C), 1618 (Amide I.), 1410.

*(1R,5S)-8-((E)-Non-2-enoyl)-8-azabicyclo[3.2.1]octan-3-one* (**3a**), yield: 107 mg (81%); colourless oil, [Anal. Calcd. for C_16_H_25_NO_2_: C, 72.97; H, 9.57; N, 5.32. Found: C, 72.76; H, 9.44; N, 5.48]; *R*_f_ (10 % EtOAc, 90 % CHCl_3_) 0.66; ^1^H NMR (500 MHz, CDCl_3_): 7.03 (1H, dt, *J* 15.0 Hz, 7.0 Hz, =*CH*), 6.23 (1 H, dt, *J* 15.0 Hz, 1.4 Hz, =*CH*), 4.97 (1 H, br s, N-*CH*), 4.60 (1 H, br s, N-*CH*), 2.85–2.75 (1 H, m, CH-*CH_a_*H_b_), 2.60–2.51 (1 H, m, CH-*CH_a_*H_b_), 2.49–2.36 (2 H, m, C(O)-CH_a_*H_b_*), 2.28–2.14 (3 H, m, overlapping of CH-CH_a_*H_b_* and =CH-*CH_2_*), 2.09 (1 H, br s, CH-CH_a_*H_b_*), 1.87–1.77 (1 H, m, C(O)-*CH_a_*H_b_), 1.75–1.65 (1H, m, C(O)-*CH_a_*H_b_), 1.52–1.42 (2 H, m, *CH_2_*CH_3_), 1.38–1.21 (6 H, br m, 3 × *CH_2_*), 0.89 (3 H, t, 7.2 Hz, CH_2_*CH_3_*); ^13^C NMR (125 MHz, CDCl_3_): 207.5, 163.7, 148.1, 119.9, 53.6, 51.4, 50.01, 48.8, 32.5, 31.6, 29.9, 28.9, 28.3, 27.8, 22.5, 14.0. MS m/z (rel. int.): 263 (9, [M]^+^), 235 (1), 206 (10), 178 (7), 150 (11), 124 (73), 81 (15), 55 (100). IR (KBr, ν (cm^−1^)): 1718 (CO), 1660 (C=C.), 1619 (Amide I.), 1425.

*(1R,5S)-8-((10S,13S)-10,13-dimethyl-2,3,4,5,6,7,8,9,10,11,12,13,14,15-tetradecahydro-1H-cyclopenta[a]phenanthrene-17-carbonyl)-8-azabicyclo[3.2.1]octan-3-one* (**4a**), yield: 163 mg (80%); pale white solid, m.p. 186–187 °C, [Anal. Calcd. for C_27_H_39_NO_2_: C, 79.17; H, 9.60; N, 3.42. Found: C, 79.13; H, 9.82; N, 3.45]; R_f_ (20 % EtOAc, 80 % CHCl_3_) 0.5; ^1^H NMR (500 MHz, CDCl_3_): 6.00 (1 H, s, =CH), 5.09/4.89 (1 H, br s, N-CH_2_), 4.62/3.56 (1 H, br s, N-CH_2_), 2.69 (2 H, br s, CH-CH_a_H_b_), 2.43 (2 H, d, 16.1 Hz, C(O)-CH_a_H_b_), 2.33–2.22 (1 H, m, steroid-CH), 2.15–2.05 (2 H, br s, CH-CH_a_H_b_), 2.01–1.92 (1 H, m, steroid-CH), 1.84–0.75 (22 H, C(O)-CH_a_H_b_ and 22 steroidal skeleton H), 1.11 (3 H, s, CH_3_), 0.85 (3 H, s, CH_3_); ^13^C NMR (125 MHz, CDCl_3_): 207.8, 166.3, 149.7, 132.3, 56.8, 55.3, 54.9, 49.8, 48.6, 47.3, 38.5, 36.5, 34.8, 33.9, 32.2, 32.1, 29.6, 29.0, 28.9, 26.8, 22.1, 20.6, 16.8, 12.2. MS m/z (rel. int.): 409 (100, [M]^+^), 394 (95), 366 (8), 285 (46), 206 (4), 161 (8), 105 (14), 67 (29). IR (KBr, ν (cm^−1^)): 1731 (CO), 1635 (C=C), 1599 (Amide I.), 1420.

*(1R,5S)-8-((1S,4R)-1,7,7-trimethylbicyclo[2.2.1]hept-2-ene-2-carbonyl)-8-azabicyclo[3.2.1]octan-3-one* (**5a**), yield: 111mg (77%); grey solid, m.p. 126–127 °C, [Anal. Calcd. for C_18_H_25_NO_2_: C, 75.22; H, 8.77; N, 4.87. Found: C, 74.61; H, 9.02; N, 5.10]; *R*_f_ (20 % EtOAc, 80 % CHCl_3_) 0.5; ^1^H NMR (500 MHz, CDCl_3_): 6.22 (1 H, s, *=CH*), 5.11/4.85 (1 H, br s, N-*CH_2_*), 4.78/3.56 (1 H, br s, N-*CH_2_*), 2.76 (2 H, br s, CH-*CH_a_*H_b_), 2.51 (1 H, dd, 4.0 Hz, 3.6 Hz, 4-*CH*), 2.46 (2 H, br s, C(O)-CH_a_*H_b_*), 2.08 (2 H, br s, CH-CH_a_*H_b_*), 2.02–1.92 (1 H, m, 5-CH_c_*CH_d_*), 1.78 (2 H, br s, C(O)-*CH_a_*H_b_), 1.66 (1 H, ddd, 12.1 Hz, 8.7 Hz, 3.7 Hz, 6-CH_c_*CH_d_*), 1.48 (1 H, ddd, 12.1 Hz, 8.9 Hz, 3.8 Hz, 6-*CH_c_*CH_d_), 1.17 (3 H, s, 9-CH_3_), 1.09 (1 H, ddd, 12.1 Hz, 8.8 Hz, 3.6 Hz, 5-*CH_c_*CH_d_), 0.89 (3 H, s, 10-CH_3_), 0.86 (3 H, s, 8-CH_3_); ^13^C NMR (125 MHz, CDCl_3_): 207.7, 166.6/, 165.3, 143.9, 136.9/136.6, 55.6, 54.5/51.1, 52.6, 50.2/49.1, 31.7, 30.1/29.6, 28.3/28.0, 25.2, 19.5, 19.1, 11.2. MS m/z (rel. int.): 287 (97, [M]^+^), 272 (47), 258 (25), 230 (8), 202 (6), 163 (100), 135 (91), 91 (75), 67 (37). IR (KBr, ν (cm^−1^)): 1721 (CO), 1620 (C=C), 1578 (Amide I.), 1427.

*(1R,5S)-8-Benzoyl-8-azabicyclo-[3.2.1]octan-3-one* (**6a**), yield: 93 mg (81%); off white solid, m.p. 108–109 °C, [Anal. Calcd. for C_14_H_15_NO_2_: C, 73.34; H, 6.59; N, 6.11. Found: C, 72.82; H, 6.45; N, 6.16]; *R*_f_ (30 % EtOAc, 70 % CHCl_3_) 0.52; ^1^H NMR (500 MHz, CDCl_3_): 7.56–7.52 (2 H, m, Ar-H), 7.51–7.41 (3 H, m, Ar-H), 5.07 (1 H, br s, N-*CH*), 4.41 (1 H, br s, N-*CH*), 2.95 (1 H, br s, CH), 2.47 (2 H, br s, CH_2_), 2.34 (1 H, br s, CH), 2.17 (2 H, br s, CH_2_), 1.77 (2 H, d, 8.2 Hz, CH_2_); ^13^C NMR (125 MHz, CDCl_3_): 207.5, 168.9, 135.5, 130.6, 128.7 (2 C), 127.1 (2 C), 56.0, 51.5, 49.7, 48.8, 29.6, 28.1. MS m/z (rel. int.): 229 (14, [M]^+^), 200 (4), 172 (7), 124 (33), 105 (100), 77 (49), 51 (13). IR (KBr, ν (cm^−1^)): 1719 (CO), 1643 (C=C), 1621 (Amide I.), 1422.

*1-((1R,5S)-3-oxo-8-azabicyclo[3.2.1]octan-8-yl)-2-phenylethane-1,2-dione* (**6a’**), yield: 57 mg (44%); off white viscous material, [Anal. Calcd. for C_15_H_15_NO_3_: C, 70.02; H, 5.88; N, 5.44.]; *R*_f_ (20 % EtOAc, 80 % CHCl_3_) 0.57; ^1^H NMR (500 MHz, CDCl_3_): 8.05 (2 H, dd, 8.4 Hz, 1.2 Hz, H*_ortho_*(Ph)), 7.69 (1 H, t, 7.4 Hz, H*_para_*(Ph)), 7.55 (2 H, t, 7.6 Hz, H*_meta_*(Ph)), 5.16–5.08 (1 H, m, N-*CH*), 4.36–4.28 (1 H, m, N-*CH*), 2.93–2.85 (2 H, m, 2 × CH), 2.54 (1 H, d, 16.4 Hz, CH), 2.42 (1 H, d, 16.4 Hz, CH), 2.26–2.08 (2 H, br m, 2 × CH), 1.87–1.79 (2 H, m, 2 × CH); ^13^C NMR (125 MHz, CDCl_3_): 206.4, 190.7, 162.3, 135.0, 132.9, 129.9 (2 C), 129.1 (2 C), 54.4, 51.2, 49.9, 49.1, 29.7, 27.9. MS m/z (rel. int.): 257 (8, [M]^+^), 152 (13), 124 (1), 105 (100), 77 (38), 67 (20), 51 (13). IR (KBr, ν (cm^−1^)): 1751 and 1718 (CO), 1653 (C=C), 1628 (Amide I.), 1452.

*(1R,5S)-8-Picolinoyl-8-azabicyclo-[3.2.1]octan-3-one* (**7a**), yield: 103 mg (90%); beige solid, m.p. 98–99 °C, [Anal. Calcd. for C_13_H_14_N_2_O_2_: C, 67.81; H, 6.13; N, 12.17. Found: C, 67.38; H, 6.13; N, 12.30]; *R*_f_ (3 % MeOH, 97 % CHCl_3_) 0.48; ^1^H NMR (500 MHz, CDCl_3_): 8.61 (1 H, ddd, 4.8 Hz, 1.7 Hz, 0.9 Hz, Py-H), 7.95 (1 H, ddd, 7.8 Hz, 1.7 Hz, 1.0 Hz, Py-H), 7.85 (1 H, td, 7.8 Hz, 1.7 Hz, Py-H), 7.41 (1 H, ddd, 7.8 Hz, 4.8 Hz, 1.2 Hz, Py-H), 5.24–5.13 (2 H, m, N-*CH*), 3.08 (1 H, dd, 16.1 Hz, 4.5 Hz, CH), 2.90 (1 H, dd, 16.1 Hz, 4.4 Hz, CH), 2.46 (2 H, q, 16.1 Hz, CH_2_), 2.24–2.10 (2 H, m, CH_2_), 1.89–1.73 (2 H, m, CH_2_); ^13^C NMR (125 MHz, CDCl_3_): 208.3, 164.3, 153.5, 148.1, 137.1, 125.3, 124.5, 55.2, 52.2, 50.3, 49.1, 30.0, 27.5. MS m/z (rel. int.): 230 (37, [M]^+^), 202 (8), 174 (8), 161 (13), 133 (4), 124 (16), 106 (36), 93 (19), 78 (100), 51 (22). IR (KBr, ν (cm^−1^)): 1718 (CO), 1629 (Amide I.), 1617 (C=C), 1586, 1472, 1448, 1420, 1411.

*(1R,5S)-8-Nicotinoyl-8-azabicyclo-[3.2.1]octan-3-one* (**8a**), yield: 94 mg (82%); yellowish oil, [Anal. Calcd. for C_13_H_14_N_2_O_2_: C, 67.81; H, 6.13; N, 12.17. Found: C, 67.52; H, 6.09; N, 12.30]; *R*_f_ (6 % MeOH, 94 % CHCl_3_) 0.45; ^1^H NMR (500 MHz, CDCl_3_): 8.78 (1 H, dd, 2.2 Hz, 0.7 Hz, Py-H), 8.69 (1 H, dd, 4.9 Hz, 1.8 Hz, Py-H), 7.87 (1 H, dt, 7.9 Hz, 1.9 Hz, Py-H), 7.38 (1 H, ddd, 7.9 Hz, 4.9 Hz, 0.8 Hz, Py-H), 5.05 (1 H, br s, N-*CH*), 4.36 (1 H, br s, N-*CH*), 2.90 (1 H, br s, CH), 2.64–2.26 (3 H, br m 3 × CH), 2.21–2.09 (2 H, m, CH_2_), 1.77 (2 H, d, 8.4 Hz, CH_2_); ^13^C NMR (125 MHz, CDCl_3_): 206.6, 166.2, 151.6, 148.0, 135.1, 131.3, 123.6, 56.1, 51.6, 49.6, 48.8, 29.7, 28.0. MS m/z (rel. int.): 230 (13, [M]^+^), 201 (3), 173 (15), 147 (2), 124 (51), 106 (100), 78 (64), 51 (28). IR (KBr, ν (cm^−1^)): 1717 (CO), 1651 (C=C), 1631 (Amide I.), 1589, 1436, 1411.

*(1R,5S)-8-(Thiophene-2-carbonyl)-8-azabicyclo[3.2.1]octan-3-one* (**9a**), yield: 96 mg (82%); beige solid, m.p. 71–72 °C, [Anal. Calcd. for C_12_H_13_NO_2_S: C, 61.25; H, 5.57; N, 5.95. Found: C, 61.04; H, 5.51; N, 6.09]; *R*_f_ (20 % EtOAc, 80 % CHCl_3_) 0.40; ^1^H NMR (500 MHz, CDCl_3_): 7.53–7.49 (2 H, m, overlapping of two thiophenyl-H), 7.09 (1 H, dd, 5.1 Hz, 3.9 Hz, thiophenyl-H), 4.99 (2 H, br s, N-*CH*), 2.80 (2 H, d, 16.1 Hz, *CH_2_*), 2.45 (2 H, d, 16.1 Hz, *CH_2_*), 2.22–2.11 (2 H, m *CH_2_*), 1.82–1.75 (2 H, m, *CH_2_*); ^13^C NMR (125 MHz, CDCl_3_): 207.2, 161.4, 137.2, 129.8, 129.5, 54.0 (2 C), 49.3 (2 C), 28.8 (2 C). MS m/z (rel. int.): 235 (11, [M]^+^), 207 (1), 178 (5), 124 (28), 111 (100), 97 (2), 83 (9), 68 (3), 54 (3). IR (KBr, ν (cm^−1^)): 1717 (CO), 1652 (C=C), 1606 (Amide I.), 1522, 1432, 1406.

*(1R,5S)-8-(1H-Indole-5-carbonyl)-8-azabicyclo[3.2.1]octan-3-one* (**10a**), yield: 91 mg (68%); off-white solid, m.p. 149–150 °C, [Anal. Calcd. for C_16_H_16_N_2_O_2_: C, 71.62; H, 6.01; N, 10.44. Found: C, 71.45; H, 5.92; N, 10.35]; *R*_f_ (99 % EtOAc, 1 % MeOH) 0.59; ^1^H NMR (500 MHz, CDCl_3_): 9.33 (1 H, br s, indole-*NH*), 7.89 (1 H, s, Ar-H), 7.37 (1 H, dd, 8.3 Hz, 1.5 Hz, Ar-H), 7.33 (1 H, d, 8.4 Hz, Ar-H), 7.23 (1 H, dd, 3.3 Hz, 2.6 Hz, Ar-H), 6.59–6.56 (1 H, m, Ar-H) 5.04 (1 H, br s, N-*CH*), 4.63 (1 H, br s, N-*CH*), 2.93 (1 H, br s, *CH*), 2.67 (1 H, br s, *CH*), 2.40 (2 H, br s, *CH_2_*), 2.26–2.14 (2 H, m *CH_2_*), 1.83–1.72 (2 H, m, *CH_2_*); ^13^C NMR (125 MHz, CDCl_3_): 208.1, 170.9, 137.1, 127.4, 126.7, 126.0, 121.1, 120.4, 111.4, 103.0, 56.2, 51.7, 49.3 (2 C), 28.7 (2 C). MS m/z (rel. int.): 268 (15, [M]^+^), 239 (1), 144 (100), 124 (4), 116 (27), 89 (10), 62 (3). IR (KBr, ν (cm^−1^)): 3318 (NH), 1706 (CO), 1629 (C=C), 1594 (Amide I.), 1431.

*Cyclohex-1-en-1-yl((1R,3R,5S)-3-hydroxy-8-azabicyclo[3.2.1]octan-8-yl)methanone* (**1b**), yield: 105 mg (89%); beige solid, m.p. 86–87 °C, [Anal. Calcd. for C_14_H_21_NO_2_: C, 71.46; H, 9.00; N, 5.95. Found: C, 71.31; H, 6.03; N, 9.18]; *R*_f_ (8 % MeOH, 92 % CHCl_3_) 0.38; ^1^H NMR (500 MHz, CDCl_3_): 5.84–5.77 (1 H, m, C=*CH*), 4.58 (1 H, br s, N-*CH*), 4.16 (1 H, br s, N-*CH*), 4.08 (1 H, t, 4.8 Hz, *CH*-OH), 3.09 (1 H, br s, OH), 2.29–1.54 (16 H, skeleton protons of nortropine and cyclohexen-1-yl ring), ^13^C NMR (125 MHz, CDCl_3_): 169.2, 134.9, 127.6, 64.6, 55.5, 50.4, 40.5, 38.7, 28.6, 27.1, 25.9, 24.6, 22.1, 21.6. MS m/z (rel. int.): 235 (39, [M]^+^), 218 (5), 207 (15), 154 (7), 126 (19), 109 (100), 81 (77), 68 (31), 53 (16). IR (KBr, ν (cm^−1^)): 3426 (OH), 1655 (C=C), 1592 (Amide I.), 1438, 1087 (C-O), 1046 γ(OH).

*(4-(tert-butyl)cyclohex-1-en-1-yl)((1R,3R,5S)-3-hydroxy-8-azabicyclo[3.2.1]octan-8-yl)methanone* (**2b**), yield: 115 mg (79%); off-white solid, m.p. 133–134 °C, [Anal. Calcd. for C_18_H_29_NO_2_: C, 74.18; H, 10.03; N, 4.81. Found: C, 73.31; H, 10.01; N, 5.16]; *R*_f_ (5 % MeOH, 95 % CHCl_3_) 0.45; ^1^H NMR (500 MHz, CDCl_3_): 5.85 (1 H, br s, C=*CH*), 4.68 (1 H, br s, N-*CH*), 4.23 (1 H, br s, N-*CH*), 4.18 (1 H, br s, *CH*-OH), 2.89 (1 H, br s, OH), 2.43–1.71 (14 H, skeleton protons of nortropine and cyclohexen-1-yl ring), 1.34–1.24 (1 H, m, Cy-*CH*), 1.23–1.13 (1 H, m, Cy-*CH*), 0.87 (9 H, C(CH_3_)_3_), ^13^C NMR (125 MHz, CDCl_3_): 169.1, 134.7, 128.1, 64.7, 55.5, 50.3, 43.5, 40.5, 38.7, 32.2, 28.7, 27.7, 27.4, 27.1, 26.5, 23.6. MS m/z (rel. int.): 291 (80, [M]^+^), 278 (24), 263 (13), 234 (51), 206 (16), 165 (77), 137 (6), 126 (38), 110 (97), 81 (65), 68 (57), 57 (100). IR (KBr, ν (cm^−1^)): 3427 (OH), 1661 (C=C), 1584 (Amide I.), 1465, 1093 (C-O), 1047 γ(OH).

*(E)-1-((1R,3R,5S)-3-hydroxy-8-azabicyclo[3.2.1]octan-8-yl)non-2-en-1-one* (**3b**), yield: 86 mg (65%); yellowish oil, [Anal. Calcd. for C_16_H_27_NO_2_: C, 72.41; H, 10.25; N, 5.28. Found: C, 71.58; H, 10.33; N, 5.33]; *R*_f_ (5 % MeOH, 95 % CHCl_3_) 0.51; ^1^H NMR (500 MHz, CDCl_3_): 6.91 (1H, dt, *J* 15.1 Hz, 7.1 Hz, =*CH*), 6.13 (1 H, dt, *J* 15.1 Hz, 1.4 Hz, =*CH*), 4.67 (1 H, br s, N-*CH*), 4.29 (1 H, br s, N-*CH*), 4.12 (1 H, t, 4.8 Hz, *CH*-OH), 2.52 (1 H, br s, OH), 2.40–1.74 (10 H, protons of nortropine skeleton and CH_2_ of octene chain), 1.50–1.39 (2 H, m, CH_2_), 1.37–1.22 (6 H, br m, 3 × *CH_2_*), 0.89 (3 H, t, 6.9 Hz, CH_2_*CH_3_*); ^13^C NMR (125 MHz, CDCl_3_): 162.7, 146.3, 120.6, 64.9, 53.9, 51.1, 40.5, 38.6, 32.4, 31.6, 28.9, 28.8, 28.4, 27.0, 22.6, 14.1. MS m/z (rel. int.): 265 (17, [M]^+^), 236 (5), 208 (9), 194 (9), 180 (25), 165 (11), 139 (26), 126 (39), 110 (47), 82 (41), 68 (77), 55 (100). IR (KBr, ν (cm^−1^)): 3409 (OH), 1658 (C=C), 1596 (Amide I.), 1448, 1091 (C-O), 1043 γ(OH).

*(((10.S,13S)-10,13-dimethyl-2,3,4,5,6,7,8,9,10,11,12,13,14,15-tetradecahydro-1H-cyclopenta[a]phenanthren-17-yl)((1R,3S,5S)-3-hydroxy-8-azabicyclo[3.2.1]octan-8-yl)methanone* (**4b**) (1:1 ratio of diastereomers), yield: 113 mg (55%); white solid, m.p. >260 °C [Anal. Calcd. for C_27_H_41_NO_2_: C, 78.78; H, 10.04; N, 3.40. Found: C, 78.26; H, 9.98; N, 3.50]; R_f_ (2 % MeOH, 98 % CHCl_3_) 0.58; ^1^H NMR (500 MHz, CDCl_3_): 5.84 (1 H, br s, =CH), 4.79/4.68 (1 H, br s, N-CH), 4.30/4.24 (1 H, br s, N-CH), 4.19 (1 H, br s, CH-OH), 2.33–0.69 (37 H, skeleton protons of steroid and nortropine + OH); ^13^C NMR (125 MHz, CDCl_3_): 165.3/165.0, 150.2/149.6, 130.5, 65.4, 56.9, 55.7/55.0, 55.4, 50.2/49.8, 48.5/48.4, 47.4, 40.8/40.5, 39.3/38.8, 38.6, 36.5, 34.9/34.3, 33.9, 32.2, 32.0/28.6, 29.1, 29.0, 27.5/27.0, 26.8, 22.2, 20.6, 16.8, 12.2. MS m/z (rel. int.): 411 (100, [M]^+^), 396 (82), 368 (4), 326 (2), 285 (20), 246 (4), 207 (12), 161 (5), 126 (10), 93 (12), 68 (10), 55 (8). IR (KBr, ν (cm^−1^)): 3355 (OH), 1613 (C=C), 1575 (Amide I.), 1435, 1096 (C-O), 1046 γ(OH).

*((1R,3S,5S)-3-hydroxy-8-azabicyclo[3.2.1]octan-8-yl)((1S,4R)-1,7,7-trimethylbicyclo[2.2.1]hept-2-en-2-yl)methanone* (**5b**) (1:1 ratio of diastereomers), yield: 91 mg (63%); white solid, m.p. 135–136 °C [Anal. Calcd. for C_18_H_27_NO_2_: C, 74.70; H, 9.40; N, 4.84. Found: C, 74.26; H, 9.36; N, 4.83]; *R*_f_ (5 % MeOH, 95 % CHCl_3_) 0.48; ^1^H NMR (500 MHz, CDCl_3_): 6.01 (1 H, s, =*CH*), 4.79/4.60 (1 H, br s, N-*CH*), 4.42/4.39 (1 H, brs, N-*CH*), 4.18 (1 H, s, *CH*-OH), 2.44 (1 H, s, OH), 2.33–1.75 (10 H, br m, CH protons of nortropine and bornene skeleton), 1.62 (1 H, ddd, 12.1 Hz, 8.5 Hz, 3.7 Hz, 6-CH_c_*CH_d_*), 1.46 (1 H, ddd, 12.1 Hz, 8.5 Hz, 3.7 Hz, 6-*CH_c_*CH_d_), 1.13 (3 H, s, 9-CH_3_), 1.06 (1 H, ddd, 12.1 Hz, 8.5 Hz, 3.7 Hz, 5-*CH_c_*CH_d_), 0.87 (3 H, s, 10-CH_3_), 0.82 (3 H, s, 8-CH_3_); ^13^C NMR (125 MHz, CDCl_3_): 165.4/164.5, 144.3/144.2, 135.2/135.1, 65.2, 55.7, 55.6/55.5, 54.4, 52.5, 50.5/49.8, 40.9/40.7, 39.2/38.6, 31.7, 29.1/28.7, 27.7/26.7, 25.3, 19.6/19.4, 19.2, 11.2. MS m/z (rel. int.): 289 (100, [M]^+^), 274 (62), 260 (28), 246 (35), 220 (3), 188 (1), 163 (77), 135 (92), 119 (32), 91 (64), 67 (27), 54 (27). IR (KBr, ν (cm^−1^)): 3425 (OH), 1617 (C=C), 1576 (Amide I.), 1443, 1088 (C-O), 1046 γ(OH).

*((1R,3R,5S)-3-hydroxy-8-azabicyclo[3.2.1]octan-8-yl)(phenyl)methanone* (**6b**), yield: 78 mg (68%); off-white solid, m.p. 126–127 °C, [Anal. Calcd. for C_14_H_17_NO_2_: C, 72.70; H, 7.41; N, 6.06. Found: C, 71.90; H, 7.27; N, 6.17]; *R*_f_ (2 % MeOH, 98 % CHCl_3_) 0.51; ^1^H NMR (500 MHz, CDCl_3_): 7.50–7.37 (5 H, m, Ar-H), 4.80 (1 H, br s, N-*CH*), 4.18 (1 H, br s, *CH*-OH), 4.06 (1 H, br s, N-*CH*), 2.37–1.71 (9 H, protons of nortropine skeleton); ^13^C NMR (125 MHz, CDCl_3_): 167.9, 136.5, 129.9, 128.4 (2 C), 127.0 (2 C), 64.9, 56.0, 51.0, 40.3, 38.7, 28.6, 27.3. MS m/z (rel. int.): 231 (20, [M]^+^), 207 (1), 186 (4), 154 (1), 127 (12), 105 (100), 77 (56), 68 (18), 51 (13). IR (KBr, ν (cm^−1^)): 3337 (OH), 1607 (C=C), 1596 (Amide I.), 1448, 1084 (C-O), 1041 γ(OH).

*1-((1R,3R,5S)-3-hydroxy-8-azabicyclo[3.2.1]octan-8-yl)-2-phenylethane-1,2-dione* (**6b’**), yield: 39 mg (30%); beige solid, m.p. 92–93 °C, [Anal. Calcd. for C_15_H_17_NO_3_: C, 69.48; H, 6.61; N, 5.40. Found: C, 69.01; H, 6.50; N, 5.47]; *R*_f_ (2 % MeOH, 98 % CHCl_3_) 0.34; ^1^H NMR (500 MHz, CDCl_3_): 7.97 (2 H, dd, 8.5 Hz, 1.2 Hz, H*_ortho_*(Ph)), 7.65 (1 H, t, 7.5 Hz, H*_para_*(Ph)), 7.51 (2 H, t, 7.5 Hz, H*_meta_*(Ph)), 4.81–4.76 (1 H, m, N-*CH*), 4.16 (1 H, t, 4.8 Hz, *CH*-OH), 3.97–3.92 (1 H, m, N-*CH*), 2.40–1.75 (9 H, skeleton protons of nortropinone); ^13^C NMR (125 MHz, CDCl_3_): 191.4, 161.7, 134.7, 133.1, 129.7 (2 C), 129.0 (2 C), 64.7, 54.6, 50.9, 40.3, 38.9, 28,5, 27.1. MS m/z (rel. int.): 259 (4, [M]^+^), 154 (85), 136 (11), 126 (1), 105 (63), 93 (100), 78 (63), 67 (38), 51 (20). IR (KBr, ν (cm^−1^)): 3424 (OH), 1681 (CO), 1620 (Amide I.), 1595 (C=C), 1451, 1082 (C-O), 1044 γ(OH), 716, 662.

*((1R,3R,5S)-3-hydroxy-8-azabicyclo[3.2.1]octan-8-yl)(pyridin-2-yl)methanone* (**7b**), yield: 85 mg (73%); beige solid, m.p. 164–165 °C, [Anal. Calcd. for C_13_H_16_N_2_O_2_: C, 67.22; H, 6.94; N, 12.06. Found: C, 66.78; H, 7.14; N, 12.16]; *R*_f_ (5 % MeOH, 95 % CHCl_3_) 0.41; ^1^H NMR (500 MHz, CDCl_3_): 8.62 (1 H, d, 4.8 Hz, Py-H), 7.86–7.79 (2 H, m, overlapping of two Py-H), 7.39 (1 H, ddd, 7.1 Hz, 4.8 Hz, 1.8 Hz, Py-H), 4.90 (1 H, brs, N-*CH*), 4.65 (1 H, brs, N-*CH*), 4.25 (1 H, t, 4.8 Hz,, *CH*-OH), 2.42–2.27 (4 H, br m, *CH_2_*), 2.11–2.00 (2 H, br m, *CH_2_*), 1.91 (1 H, d, 14.6 Hz, *CH*), 1.87–1.73 (overlapping, 1 H, d, 14.6 Hz, *CH* and 1H of OH); ^13^C NMR (125 MHz, CDCl_3_): 163.9, 154.3, 148.2, 136.9, 124.7, 123.7, 64.9, 55.3, 51.7, 40.5, 38.9, 28.7, 26.7. MS m/z (rel. int.): 232 (46, [M]^+^), 216 (13), 187 (2), 160 (17), 126 (63), 106 (36), 78 (100), 66 (18), 51 (20). IR (KBr, ν (cm^−1^)): 3354 (OH), 1599 (Amide I.), 1579 (C=C), 1536, 1479, 1447, 1410, 1089 (C-O), 1038 γ(OH).

*((1R,3R,5S)-3-hydroxy-8-azabicyclo[3.2.1]octan-8-yl)(pyridin-3-yl)methanone* (**8b**), yield: 72 mg (62%); yellowish solid, m.p. 113–114 °C, [Anal. Calcd. for C_13_H_16_N_2_O_2_: C, 67.22; H, 6.94; N, 12.06. Found: C, 66.83; H, 6.69; N, 12.13]; *R*_f_ (5 % MeOH, 95 % CHCl_3_) 0.32; ^1^H NMR (500 MHz, CDCl_3_): 8.71 (1 H, d, 1.4 Hz, Py-H), 8.67 (1 H, dd, 5.0 Hz, 1.4 Hz, Py-H), 7.82 (1 H, dt, 7.8 Hz, 2.0 Hz, Py-H), 7.38 (1 H, ddd, 7.8 Hz, 4.8 Hz. 0.8 Hz, Py-H), 4.81 (1 H, br s, N-*CH*), 4.21 (1 H, t, 4.8 Hz, *CH*-OH), 4.03 (1 H, br s, N-*CH*), 2.45–1.77 (9 H, protons of nortropine skeleton and OH); ^13^C NMR (125 MHz, CDCl_3_): 165.0, 150.8, 147.8, 135.0, 132.2, 123.5, 64.7, 56.3, 51.3, 40.5, 38.7, 28.7, 27.2. MS m/z (rel. int.): 232 (24, [M]^+^), 213 (1), 187 (6), 154 (2), 126 (18), 106 (100), 78 (64), 66 (22), 51 (27). IR (KBr, ν (cm^−1^)): 3422 (OH), 1612 (Amide I.), 1591 (C=C), 1443 (C-N), 1412, 1088 (C-O), 1046 γ(OH).

*((1R,3R,5S)-3-hydroxy-8-azabicyclo[3.2.1]octan-8-yl)(thiophen-2-yl)methanone* (**9b**), yield: 72 mg (92%); brownish oil, [Anal. Calcd. for C_12_H_15_NO_2_S: C, 60.73; H, 6.37; N, 5.90. Found: C, 59.98; H, 6.33; N, 5.98]; *R*_f_ (5 % MeOH, 95 % CHCl_3_) 0.49; ^1^H NMR (500 MHz, CDCl_3_):7.47 (1 H, dd, 5.0 Hz, 1.1 Hz, Ar-H), 7.44 (1 H, dd, 3.7 Hz, 1.1 Hz, Ar-H), 7.08 (1 H, dd, 5.3 Hz, 3.7 Hz, Ar-H), 5.00–4.51 (2 H, overlapping of two br s, N-*CH*), 4.24 (1 H, t, 4.8 Hz, *CH*-OH), 2.37–2.28 (2 H, m, *CH_2_*), 2.25 (1 H, br s, OH), 2.06–1.99 (2 H, m, *CH_2_*), 1.91 (2 H, d, 14.7 Hz, *CH_2_*), 1.69 (2 H, s, *CH_2_*); ^13^C NMR (125 MHz, CDCl_3_): 160.2, 138.0, 129.0, 128.7, 126.9, 64.7, 55.9, 52.0, 40.2, 36.9, 28.8, 26.7. MS m/z (rel. int.): 237 (17, [M]^+^), 218 (1), 192 (1), 154 (1), 126 (10), 111 (100), 83 (10), 66 (15), 51 (1). IR (KBr, ν (cm^−1^)): 3424 (OH), 1587 (Amide I.), 1522 (C=C), 1443, 1087 (C-O), 1045 γ(OH).

*((1R,3R,5S)-3-hydroxy-8-azabicyclo[3.2.1]octan-8-yl)(1H-indol-5-yl)methanone* (**10b**), yield: 69 mg (51%); white solid, m. p: 92–93 °C, [Anal. Calcd. for C_16_H_18_N_2_O_2_: C, 71.09; H, 6.71; N, 10.36. Found: C, 70.95; H, 6.58; N, 10.19]; *R*_f_ (5 % MeOH, 95 % CHCl_3_) 0.49; ^1^H NMR (500 MHz, DMSO-*d*_6_): 11.29 (1 H, br s, NH), 7.67 (1 H, s, Ar-H), 7.44–7.39 (2 H, m, Ar-H), 7.19 (1 H, dd, 8.4 Hz and 1.4 Hz, Ar-H), 6.50 (1 H, s, Ar-H), 4.62 (1 H, d, 2.4 Hz, *CH*-OH), 4.55 (1 H, br s, N-*CH*), 4.10 (1 H, br s, N-*CH*), 2.25 (1 H, br s, OH), 2.30–1.55 (8 H, protons of nortropine skeleton); ^13^C NMR (125 MHz, DMSO-*d*_6_): 168.9, 137.0, 127.7, 127.4, 126.9, 121.0, 120.0, 111.5, 102.3, 63.6, 56.5, 51.2, 39.2 (overlaping with DMSO-*d*_6_), 38.4, 28.7, 27.5. MS m/z (rel. int.): 270 (19, [M]^+^), 207 (1), 160 (11), 144 (100), 116 (25), 89 (10), 62 (2). IR (KBr, ν (cm^−1^)): 3414 (OH), 3257 (NH), 1590 (Amide I.), 1571 (C=C), 1440, 1324, 1086 (C-O), 1045 γ(OH).

## 4. Conclusions

In summary, nortropane-based nucleophiles (nortropinone (**a**), nortropine (**b**)) can be used as *N*-nucleophiles in palladium-catalyzed aminocarbonylation. In the presence of simple iodoalkenes, as well as biologically important skeletons possessing iodoalkene functionality, the target carboxamide derivatives have been produced exclusively by using Pd(OAc)_2_/PPh_3_ catalysts. The iodobenzene has shown lower reactivity than the iodoalkene substrates using the above-mentioned catalysts. Thus, increasing the carbon monoxide pressure (40 bar), the chemoselectivity has been shifted toward the corresponding 2-ketocarboxamide formed due to the double CO insertion, but the conversion was still not complete after 48 h reaction time. Changing the triphenylphosphine to the bidentate Xantphos, the target carboxamide has been formed selectively under mild reaction conditions (1 bar of CO, 50–70 °C). Using these optimized reaction conditions, we have been able to synthesize various *N*-acylnortropane derivatives in the presence of iodo-(hetero)arenes in aminocarbonylation reactions. The new carboxamide derivatives were isolated in moderate to good yields (51–92%), and they were fully characterized.

It can be stated that the palladium-catalyzed aminocarbonylation provides an efficient tool for the “acylation” of amines possessing biologically important skeletons. It is based on the good acylating ability of the palladium(II)-acyl species formed during the catalytic cycle of the aminocarbonylation. In this way, important carboxamides could be synthesized that cannot be produced by using conventional organic synthetic methods.

## Figures and Tables

**Table 1:** Aminocarbonylation of nortropane-based nucleophiles (**a**, **b**) in the presence of iodoalkenes (**1**–**5**) using Pd(OAc)_2_ +2 PPh_3_ in situ catalyst ^a^.

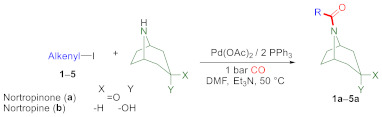					
Entry	Substrate	Amine	R. Time [h]	Conv. ^b^ [18]	Isolated Yield ^c^ [18]
1	1-iodocyclohexene (**1**)	**a**	4	3	n.i.
2	1-iodocyclohexene (**1**)	**a**	24	42	n.i.
3	1-iodocyclohexene (**1**)	**a**	48	88	n.i.
4	1-iodocyclohexene (**1**) ^d^	**a**	4	70	n.i.
5	1-iodocyclohexene (**1**) ^d^	**a**	24	100	80 (**1a**)
6	4-*tert*-butyl-1-iodocyclohexene (**2**)	**a**	4	100	86 (**2a**)
7	*trans*-1-iodo-1-octene (**3**)	**a**	4	100	81 (**3a**)
8	17-iodoandrost-16-ene (**4**)	**a**	24	100	80 (**4a**)
9	2-iodobornene (**5**)	**a**	4	75	n.i.
10	2-iodobornene (**5**)	**a**	24	100	77 (**5a**)
11	1-iodocyclohexene (**1**)	**b**	1	100	89 (**1b**)
12	4-*tert*-butyl-1-iodocyclohexene (**2**)	**b**	1	100	79 (**2b**)
13	*trans*-1-iodo-1-octene (**3**)	**b**	1	100	65 (**3b**)
14	17-iodoandrost-16-ene (**4**)	**b**	1	80	n.i.
15	17-iodoandrost-16-ene (**4**)	**b**	6	100	55 (**4b**)
16	2-iodobornene (**5**)	**b**	1	100	63 (**5b**)

^a^ Reaction conditions: 0.5 mmol substrate (**1**–**5**), 0.55 mmol of nucleophile (**a**, **b**), 0.025 mmol of Pd(OAc)_2_, 0.05 mmol of PPh_3_, 0.5 mL of Et_3_N, 10 mL of DMF, 50 °C, 1 bar of CO. The reaction was purged with Argon and then replaced by carbon monoxide. ^b^ Determined by GC-MS. ^c^ Yields of the isolated target compound (based on the substrates (**1**–**5**)); n.i. = not isolated. ^d^ The reaction was carried out at 40 bar carbon monoxide pressure.

**Table 2:** Aminocarbonylation of nortropane-based nucleophiles (**a**, **b**) in the presence of iodo-heteroaromatics (**7**–**10**) using Pd(OAc)_2_ + Xantphos in situ catalyst ^a^.

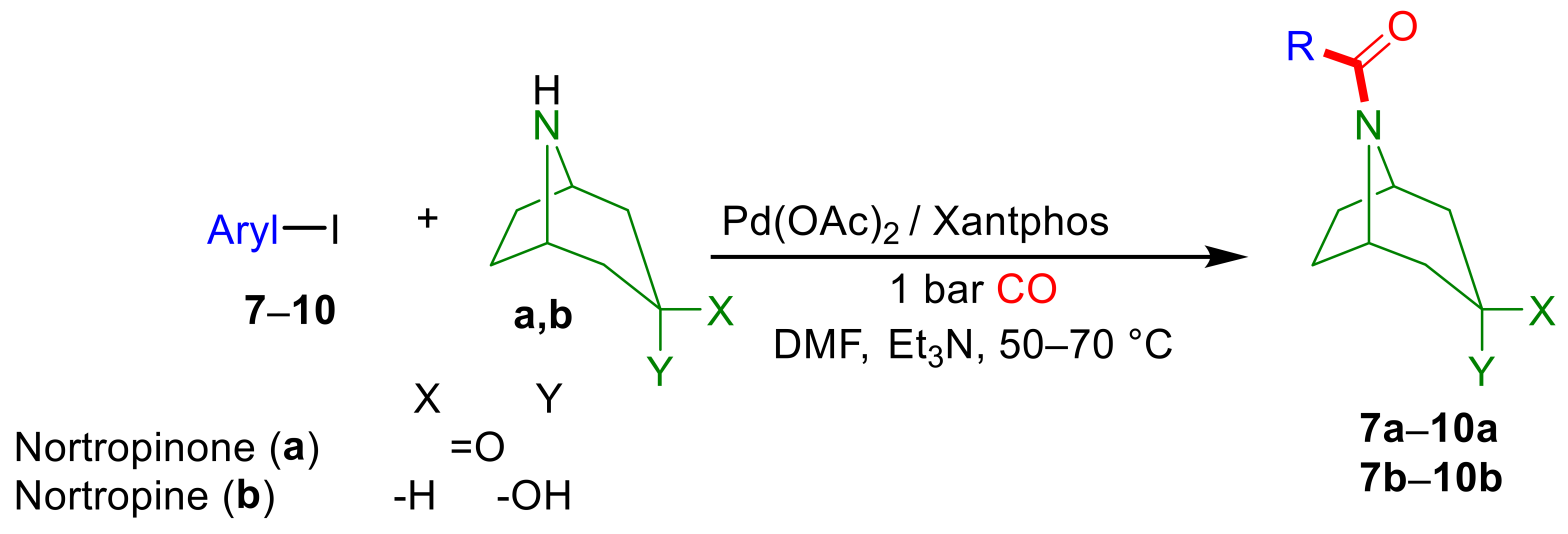						
Entry	Substrate	Amine	R. Time [h]	Temp [°C]	Conv. ^b^ [18]	Isolated Yield ^c^ [18]
1	2-Iodopyridine (**7**)	**a**	6	70	100	90 (**7a**)
2	3-Iodopyridine (**8**)	**a**	6 ^d^	70	66	n.i.
3	3-Iodopyridine (**8**)	**a**	24	70	100	82 (**8a**)
4	2-Iodothiophene (**9**)	**a**	6 ^d^	70	82	n.i.
5	2-Iodothiophene (**9**)	**a**	24	70	100	82 (**9a**)
6	5-Iodoindole (**10**)	**a**	6 ^d^	70	47	n.i.
7	5-Iodoindole (**10**)	**a**	24 ^d^	70	68	n.i.
8	5-Iodoindole (**10**)	**a**	48	70	86	68 (**10a**)
9	2-Iodopyridine (**7**)	**b**	2	50	100	73 (**7b**)
10	3-Iodopyridine (**8**)	**b**	2	50	100	62 (**8b**)
11	2-Iodothiophene (**9**)	**b**	2 ^d^	50	85	n.i.
12	2-Iodothiophene (**9**)	**b**	6	50	100	92 (**9b**)
13	5-Iodoindole (**10**)	**b**	2 ^d^	50	90	n.i.
14	5-Iodoindole (**10**)	**b**	4	50	100	51 (**10b**)

^a^ Reaction conditions: 0.5 mmol substrate (**7**–**10**), 0.55 mmol of nucleophile (**a**, **b**), 0.025 mmol of Pd(OAc)_2_, 0.025 mmol of Xantphos, 0.5 mL of Et_3_N, 10 mL of DMF, 1 bar of CO. The reaction was purged with argon and then replaced by carbon monoxide. ^b^ Determined by GC–MS. ^c^ Yields of the isolated target compound (based on the substrates (**7**–**10**)); n.i. = not isolated. ^d^ Sample was taken and analyzed by GC.

## Data Availability

The data presented in this study are available in the Supplementary Materials.

## Supplementary Materials

^1^H and ^13^C NMR spectra of the products synthesized in this work are available online.

## Sample Availability

Samples of the compounds are not available from the authors.
