# Supplementary material for: Selective Synthesis of N-Acylnortropane Derivatives in Palladium-Catalysed Aminocarbonylation
Source: Molecules. 2021 Mar 23;26(6):1813. doi: 10.3390/molecules26061813 (PMC8004868; doi:10.3390/molecules26061813)

Selective Synthesis of *N*-Acylnortropane Derivatives in Palladium-Catalysed Aminocarbonylation.

László Kollár [1, 2, 3], Ádám Erdélyi [1], Haroon Rasheed [1] and Attila Takács [1, 2, *]

[1] Department of Inorganic Chemistry, Faculty of Sciences, University of Pécs, Ifjúság útja. 6., H-7624 Pécs, Hungary; kollar@gamma.ttk.pte.hu (L.K.); erdelyi.adam99@gmail.com (Á.E.); privateharoon@gmail.com (H.R.)
[2] János Szentágothai Research Centre, University of Pécs, Ifjúság útja. 20., H-7624 Pécs, Hungary
[3] MTA-PTE Research Group for Selective Chemical Syntheses, Ifjúság útja. 6., H-7624 Pécs, Hungary
* Correspondence: takacsattila@gamma.ttk.pte.hu; Tel.: +36-72-503-600

Supplementary Material

Content

^{1}H and ^{13}C NMR spectra of the *N*-acylnortropane derivatives (**1a-10a, 1b-10b**) p2-p23

- *^{1}H and ^{13}C NMR spectra of* ***1a*** *p2*
- *^{1}H and ^{13}C NMR spectra of* ***2a*** *p3*
- *^{1}H and ^{13}C NMR spectra of* ***3a*** *p4*
- *^{1}H and ^{13}C NMR spectra of* ***4a*** *p5*
- *^{1}H and ^{13}C NMR spectra of* ***5a*** *p6*
- *^{1}H and ^{13}C NMR spectra of* ***6a*** *p7*
- *^{1}H and ^{13}C NMR spectra of* ***6a'*** *p8*
- *^{1}H and ^{13}C NMR spectra of* ***7a*** *p9*
- *^{1}H and ^{13}C NMR spectra of* ***8a*** *p10*
- *^{1}H and ^{13}C NMR spectra of* ***9a*** *p11*
- *^{1}H and ^{13}C NMR spectra of* ***10a*** *p12*
- *^{1}H and ^{13}C NMR spectra of* ***1b*** *p13*
- *^{1}H and ^{13}C NMR spectra of* ***2b*** *p14*
- *^{1}H and ^{13}C NMR spectra of* ***3b*** *p15*
- *^{1}H and ^{13}C NMR spectra of* ***4b*** *p16*
- *^{1}H and ^{13}C NMR spectra of* ***5b*** *p17*
- *^{1}H and ^{13}C NMR spectra of* ***6b*** *p18*
- *^{1}H and ^{13}C NMR spectra of* ***6b'*** *p19*
- *^{1}H and ^{13}C NMR spectra of* ***7b*** *p20*
- *^{1}H and ^{13}C NMR spectra of* ***8b*** *p21*
- *^{1}H and ^{13}C NMR spectra of* ***9b*** *p22*
- *^{1}H and ^{13}C NMR spectra of* ***10b*** *p23*

^1H and ^{13}C NMR spectra of **1a**

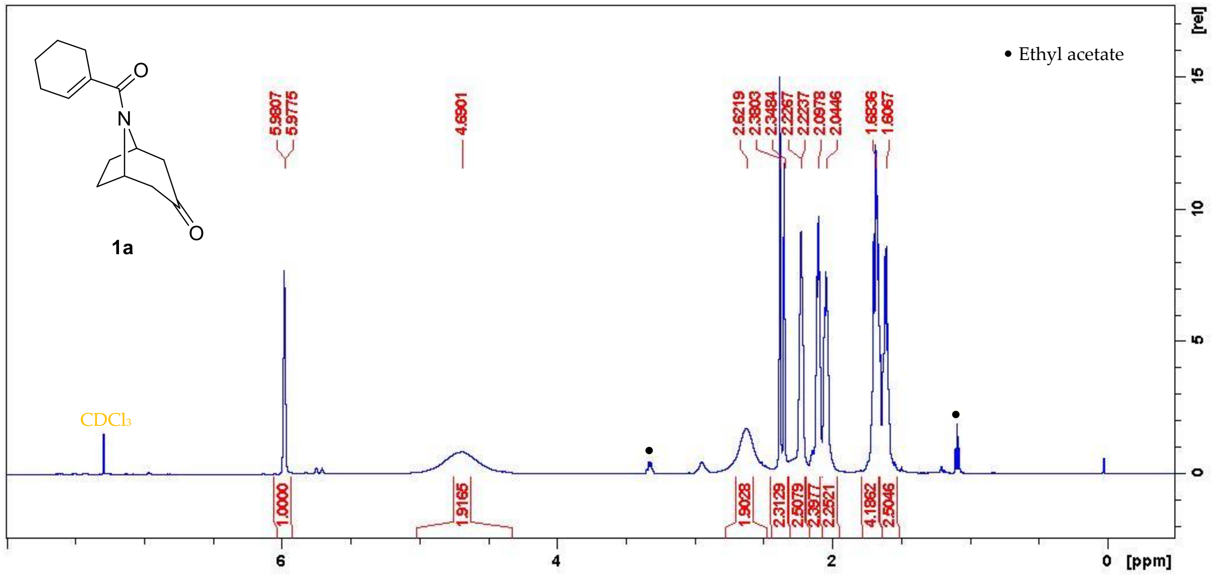

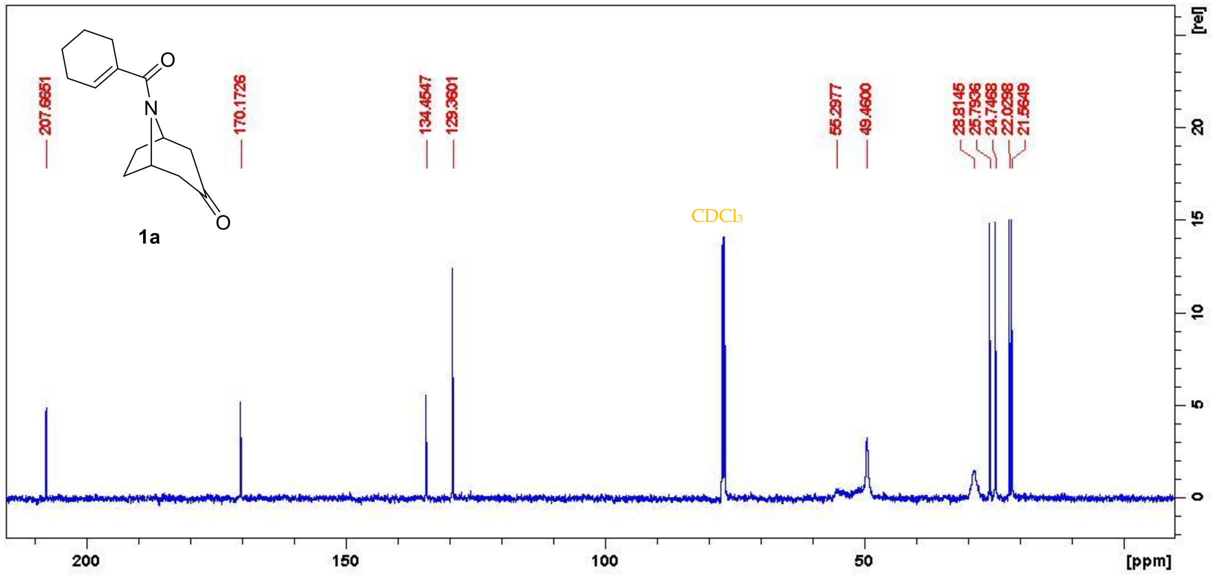

S

^{1}H and ^{13}C NMR spectra of **2a**

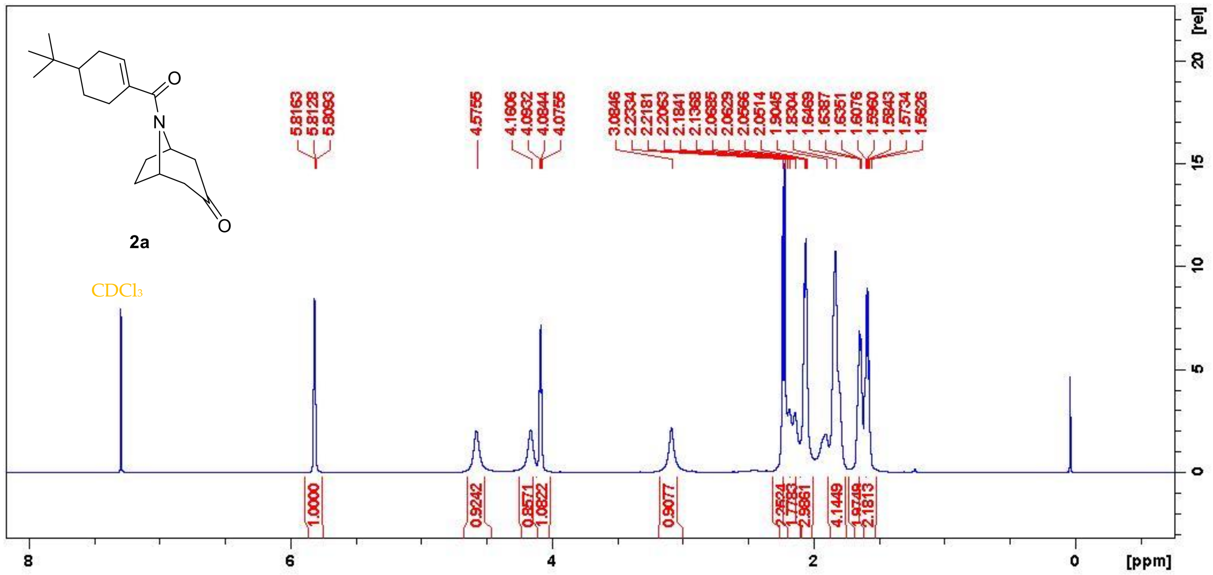

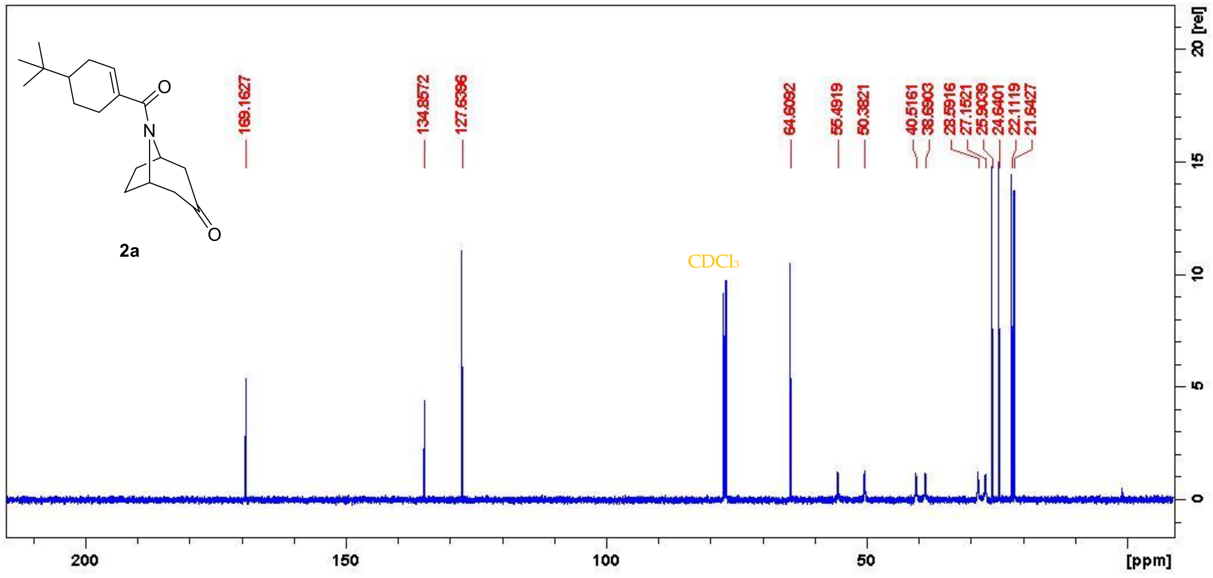

^{1}H and ^{13}C NMR spectra of **3a**

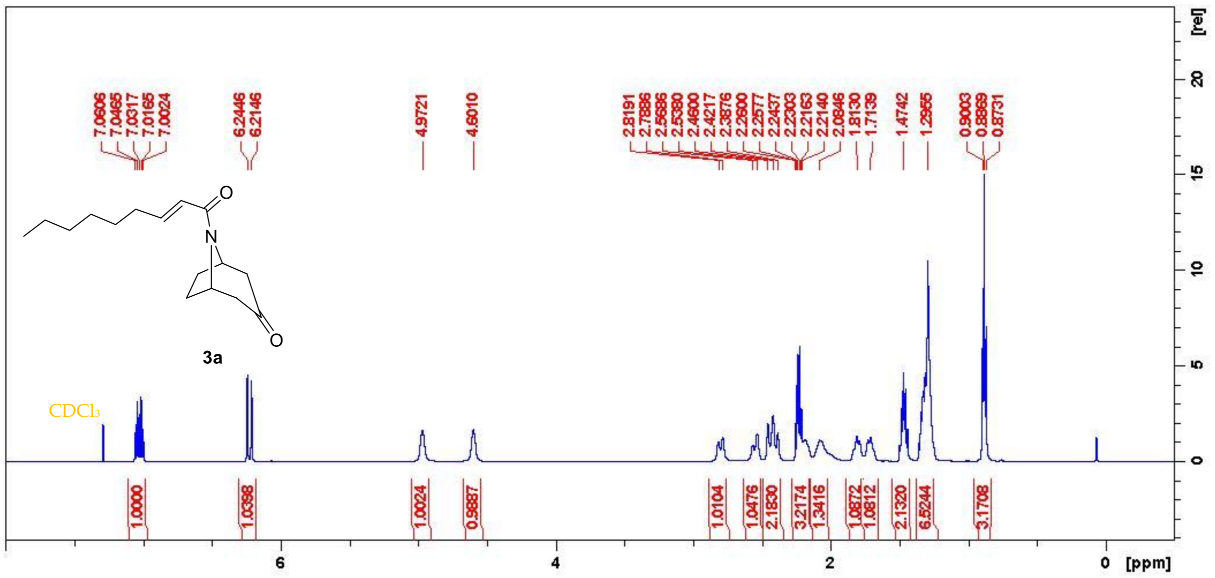

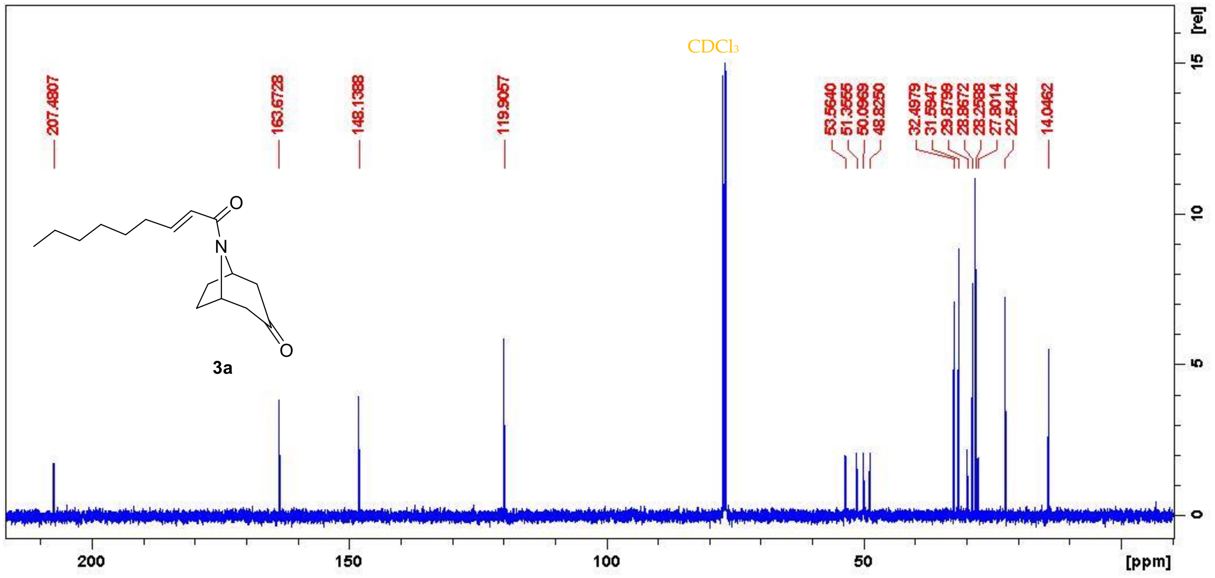

^{1}H and ^{13}C NMR spectra of **4a**

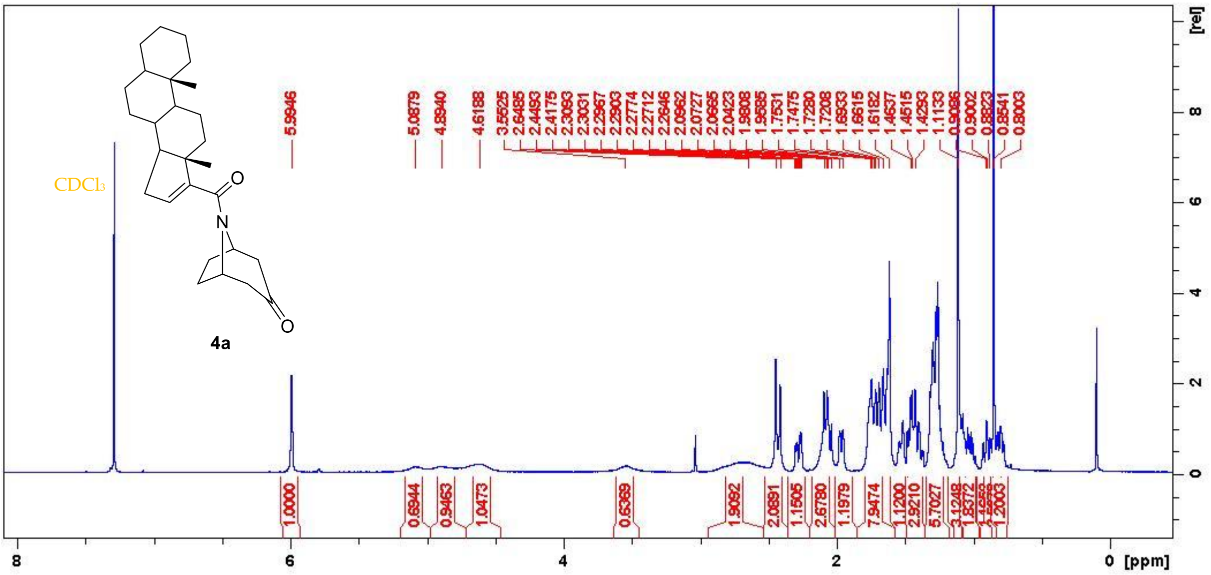

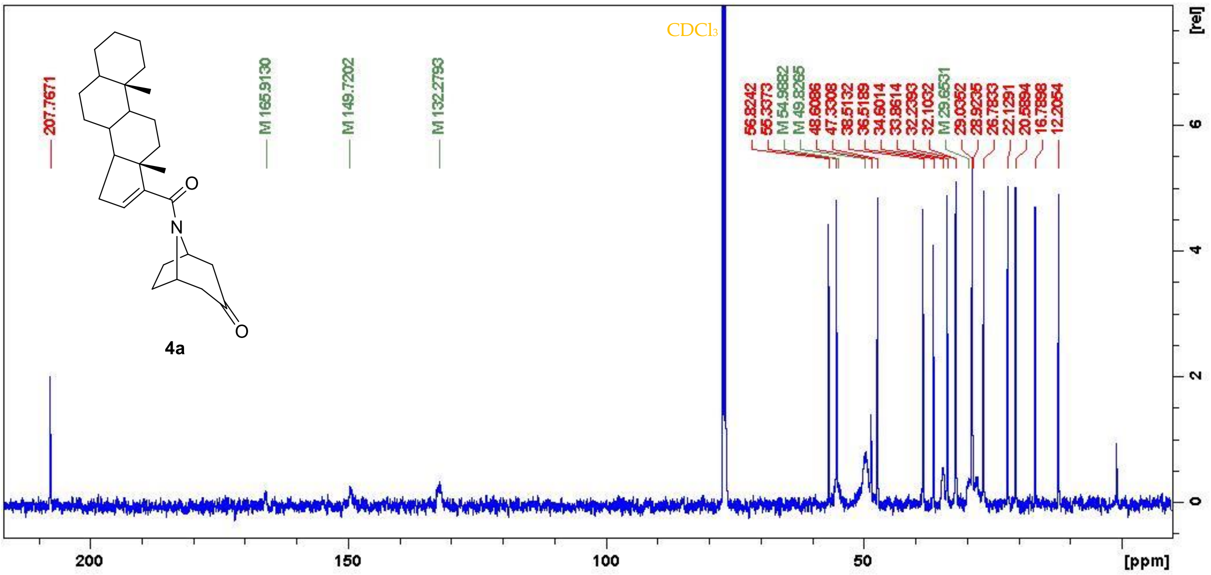

^{1}H and ^{13}C NMR spectra of **5a**

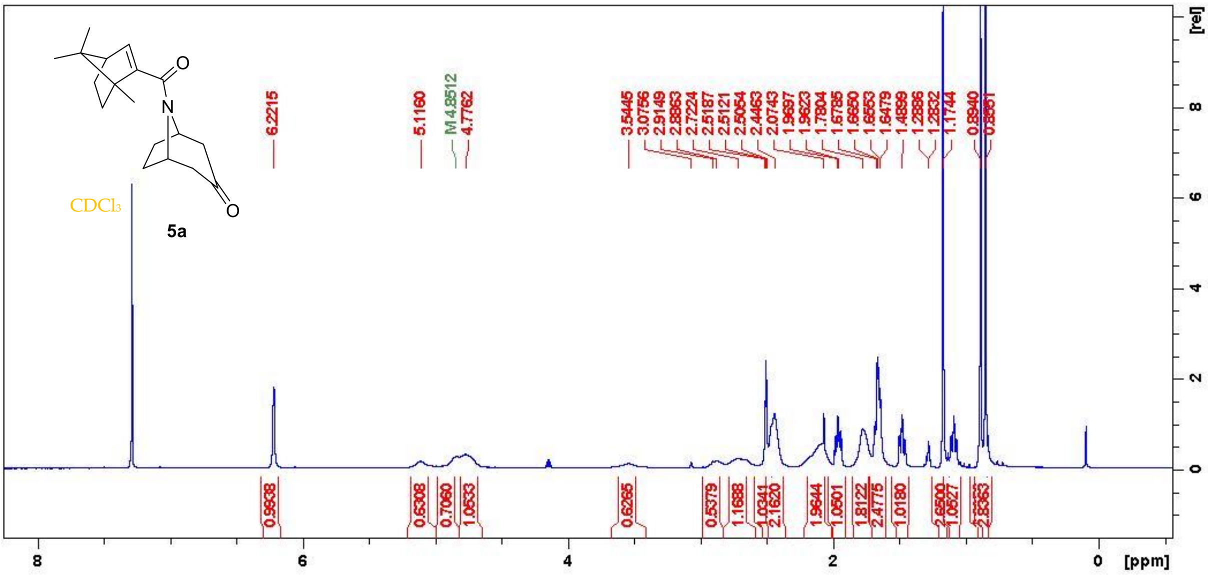

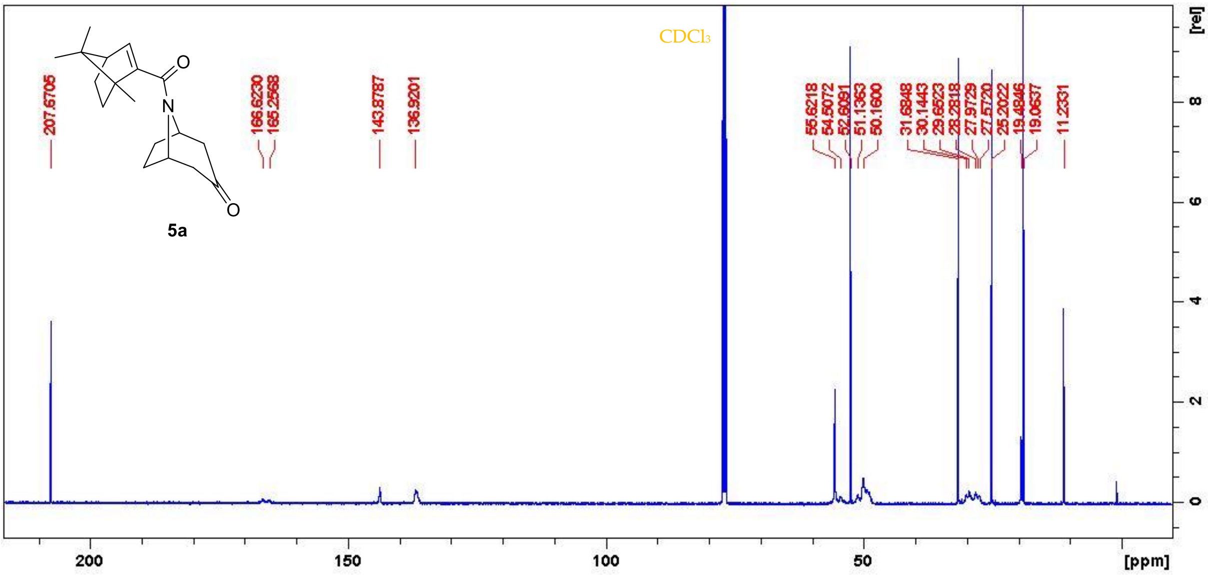

^{1}H and ^{13}C NMR spectra of **6a**

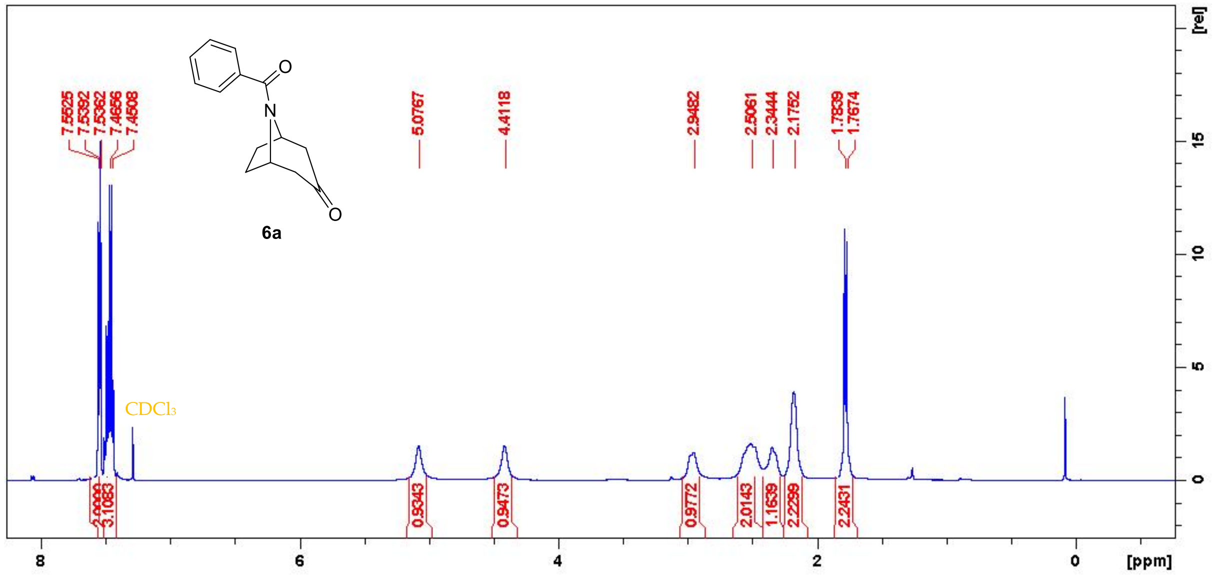

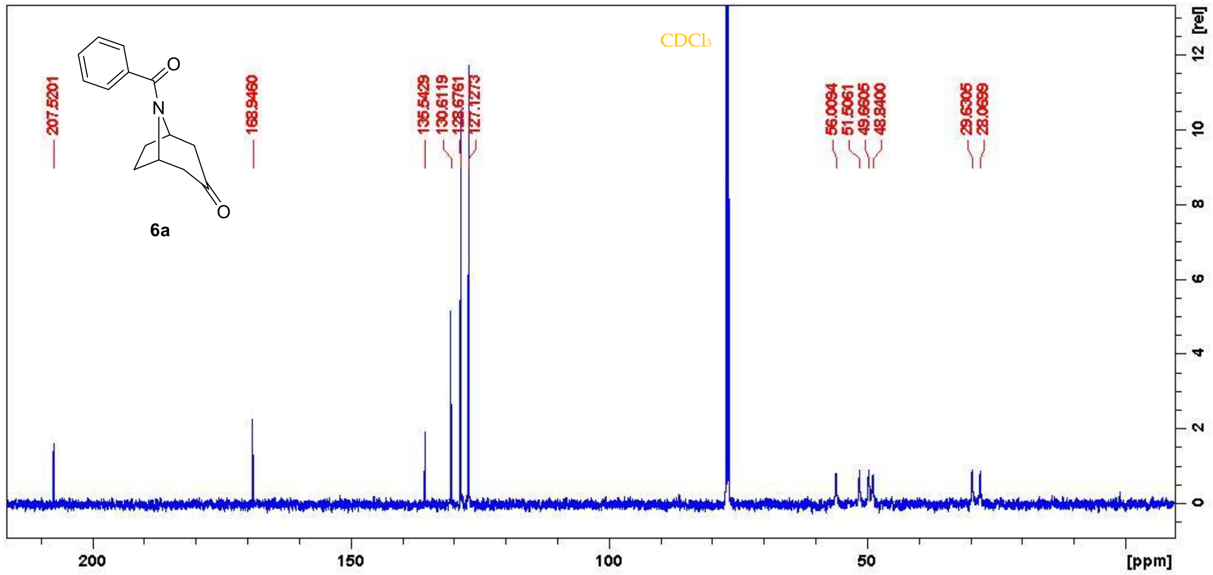

^{1}H and ^{13}C NMR spectra of **6a′**

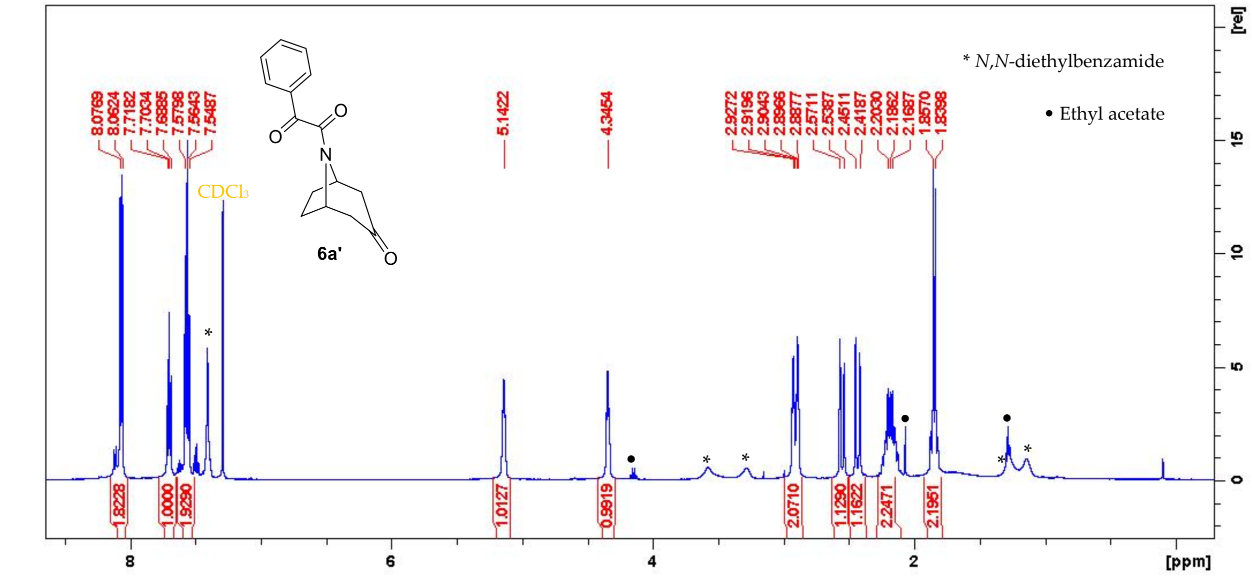

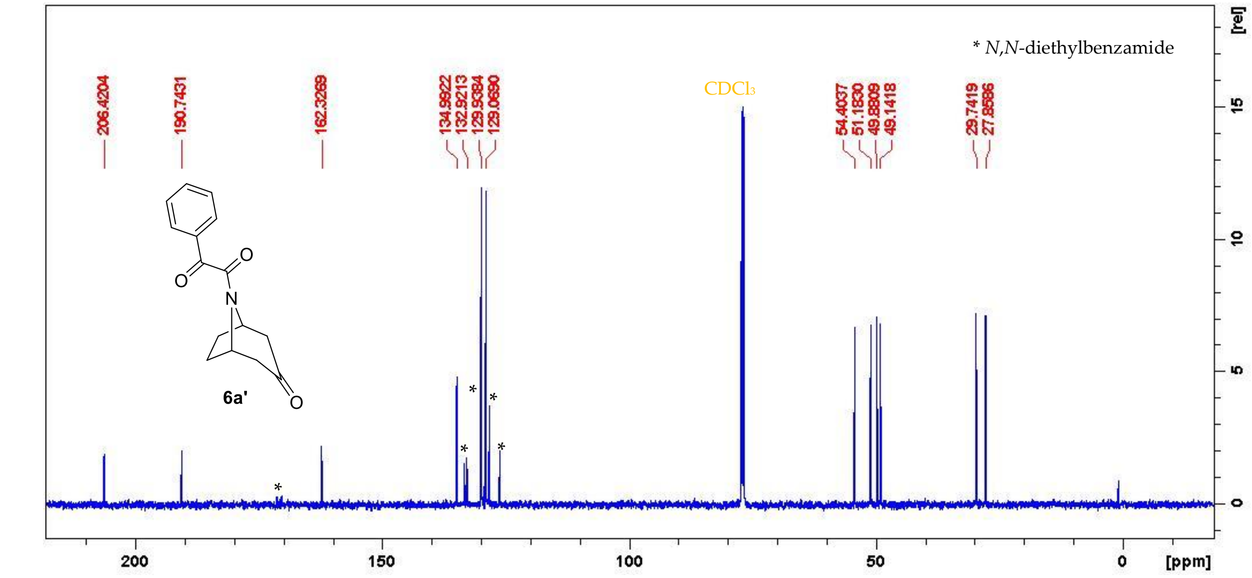

^{1}H and ^{13}C NMR spectra of **7a**

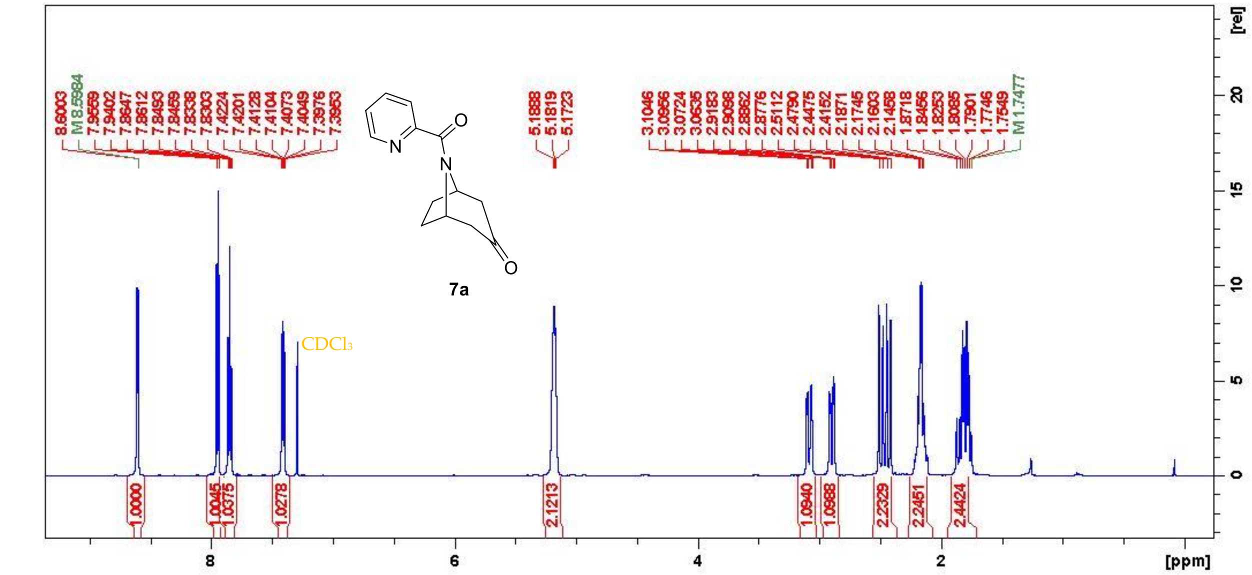

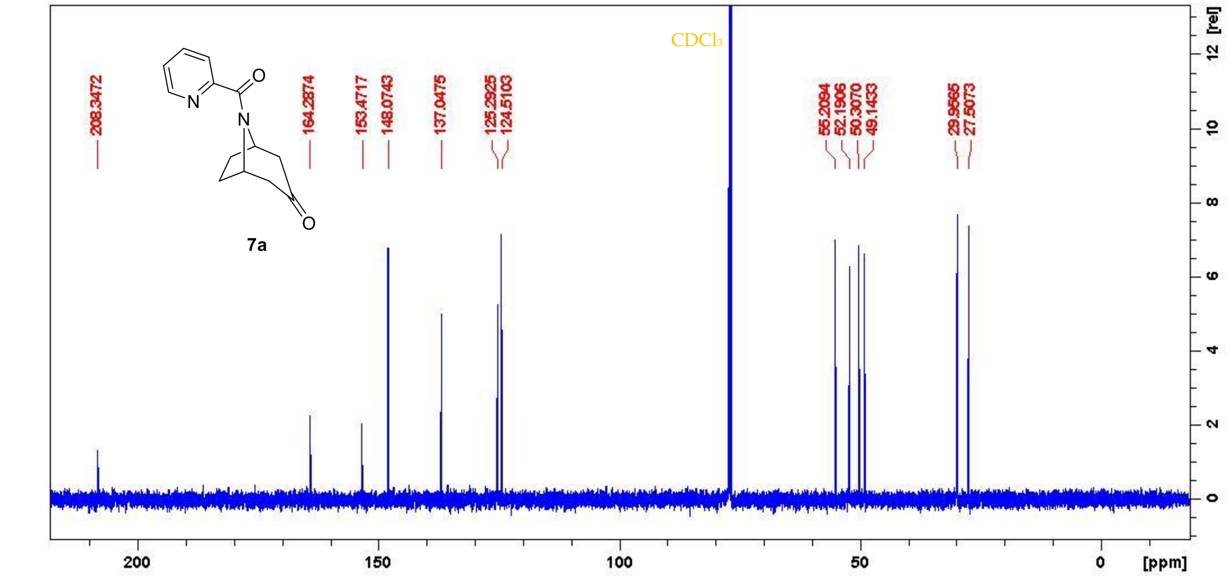

^{1}H and ^{13}C NMR spectra of **8a**

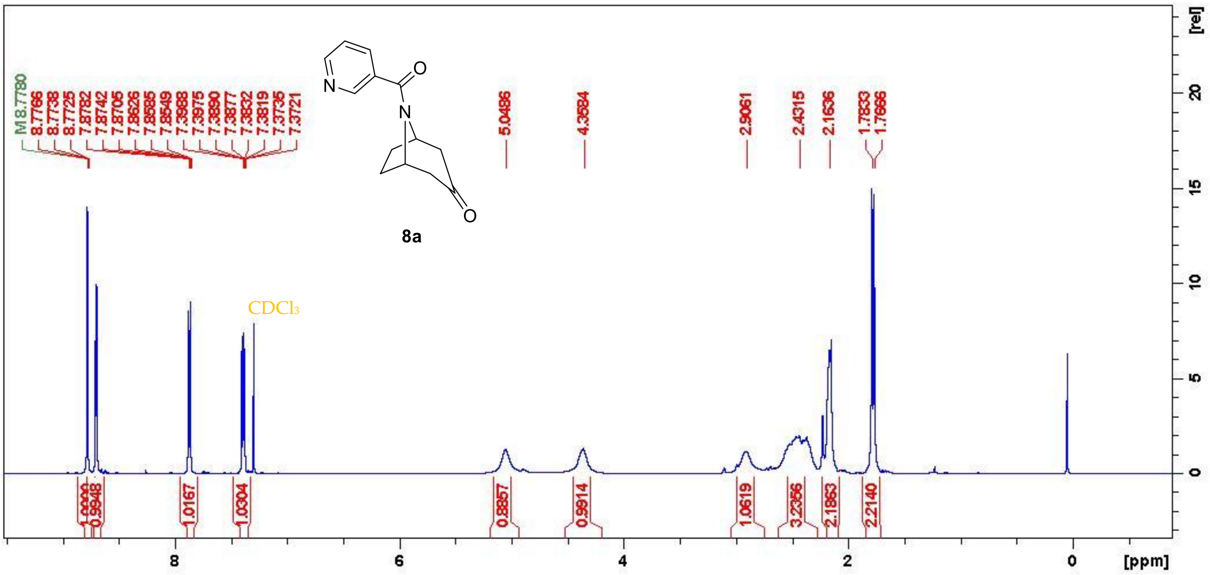

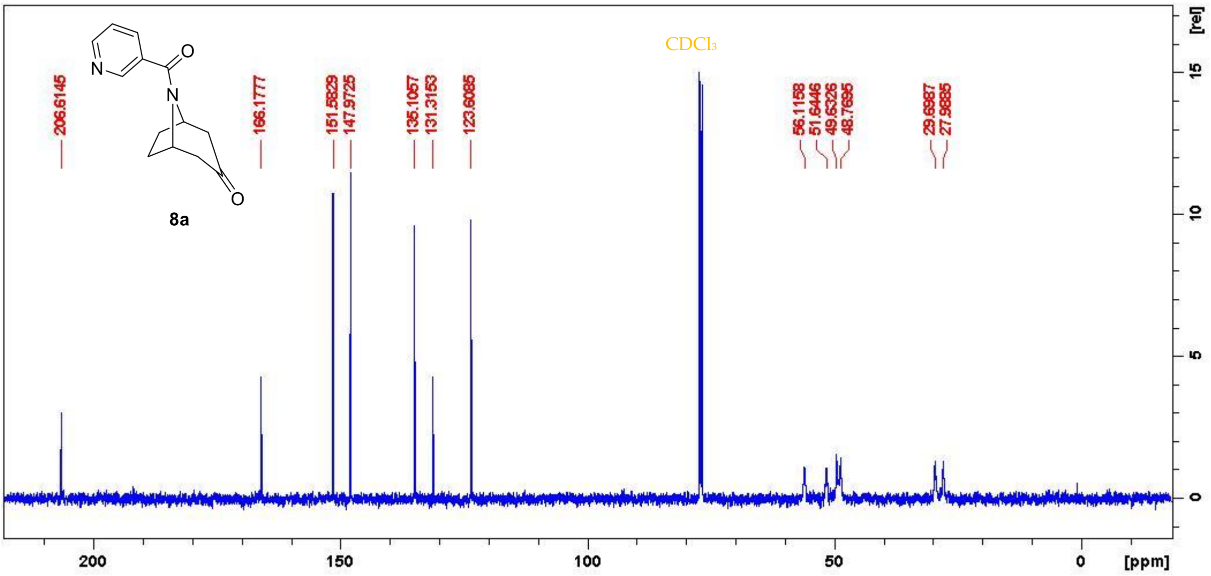

^{1}H and ^{13}C NMR spectra of **9a**

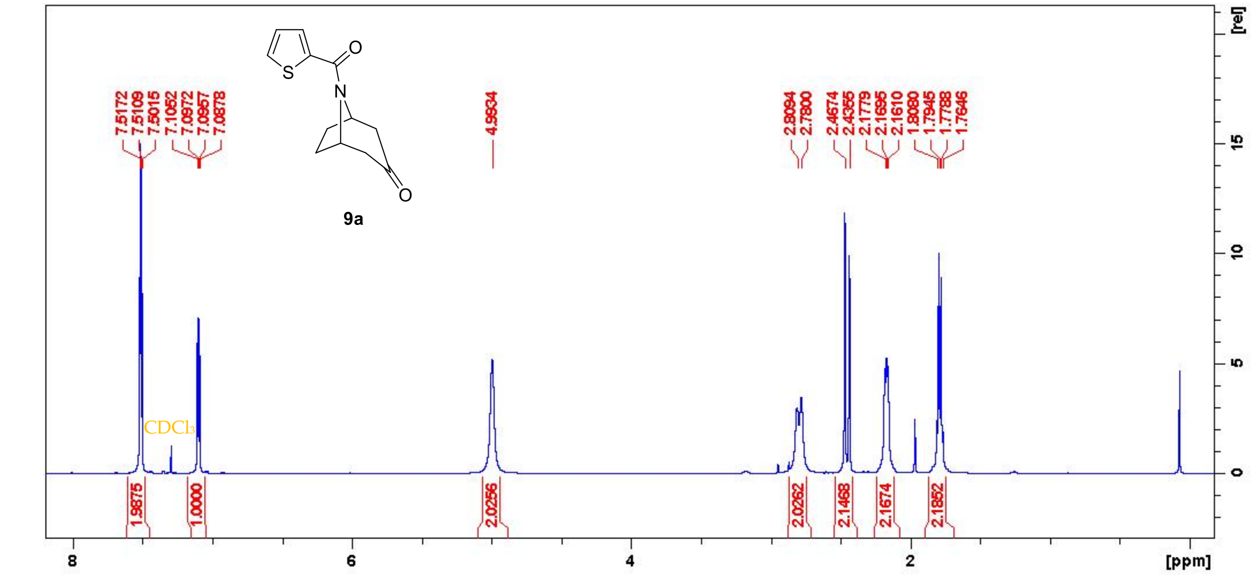

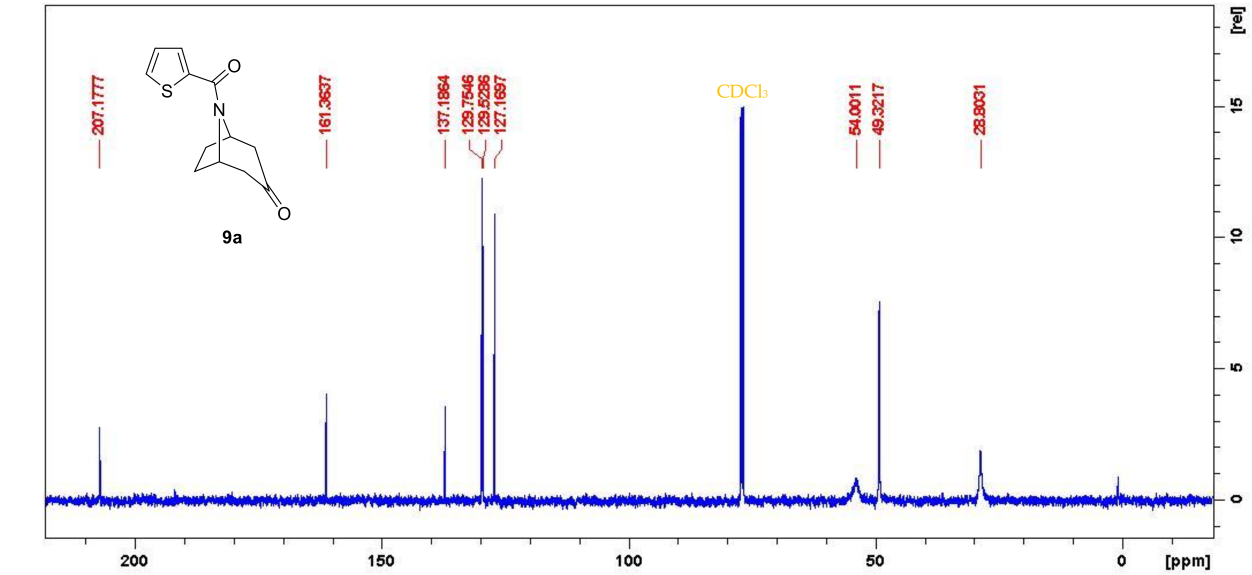

^{1}H and ^{13}C NMR spectra of **10a**

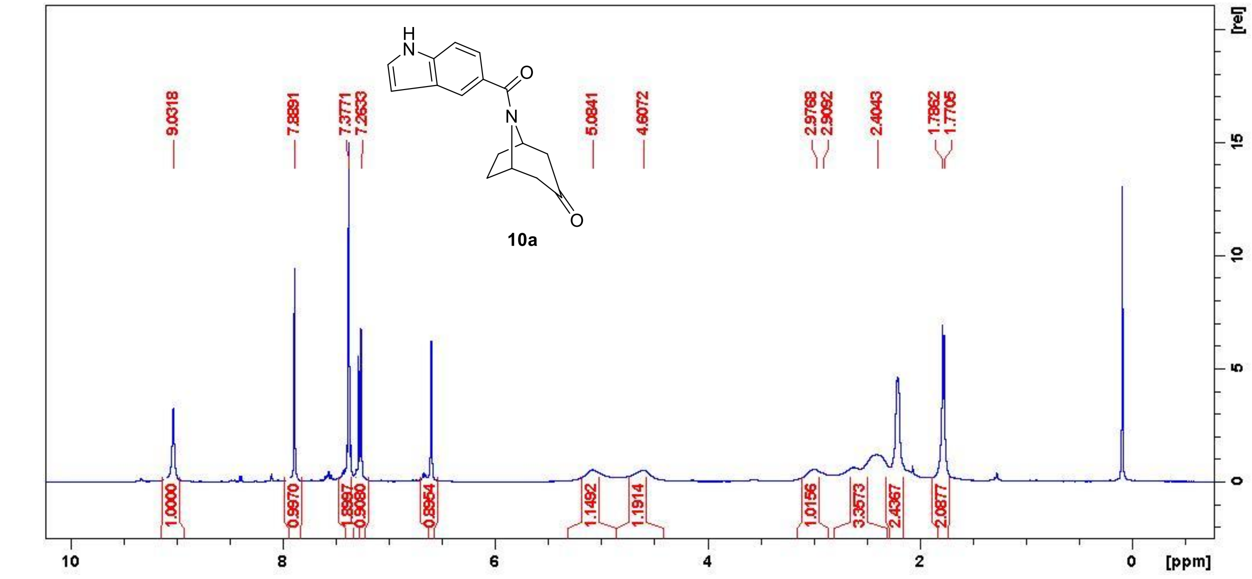

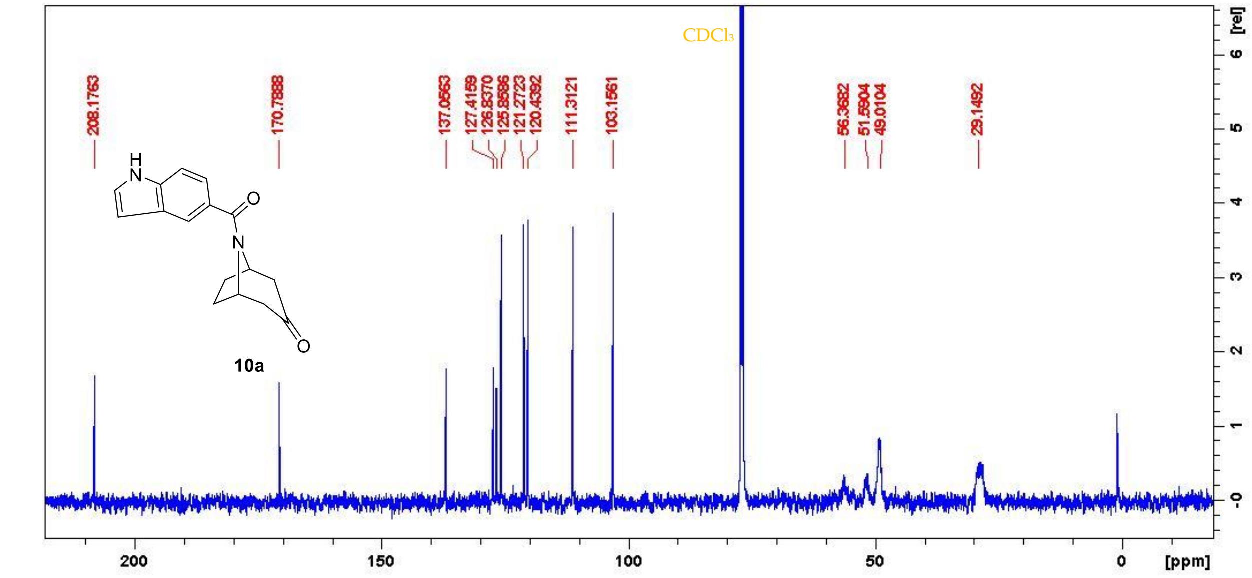

^{1}H and ^{13}C NMR spectra of **1b**

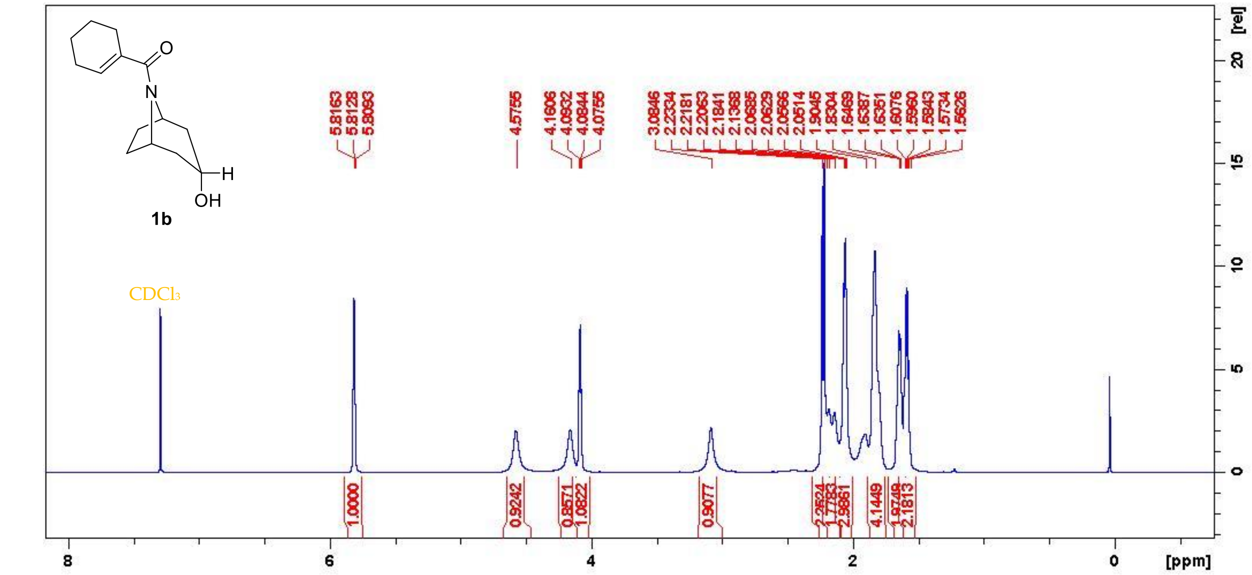

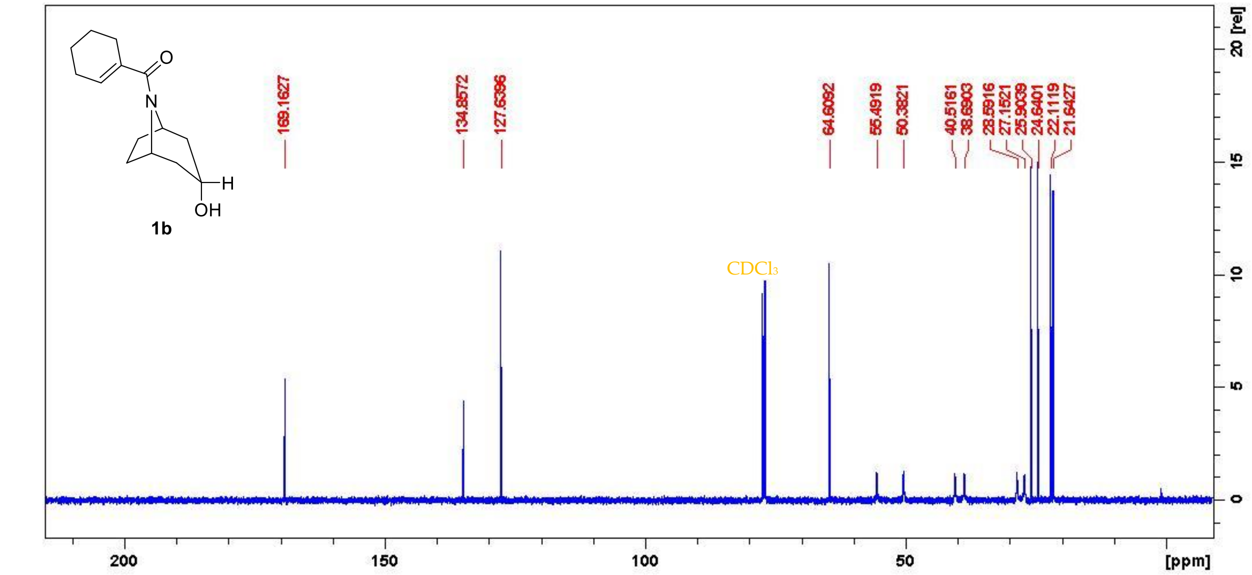

^{1}H and ^{13}C NMR spectra of **2b**

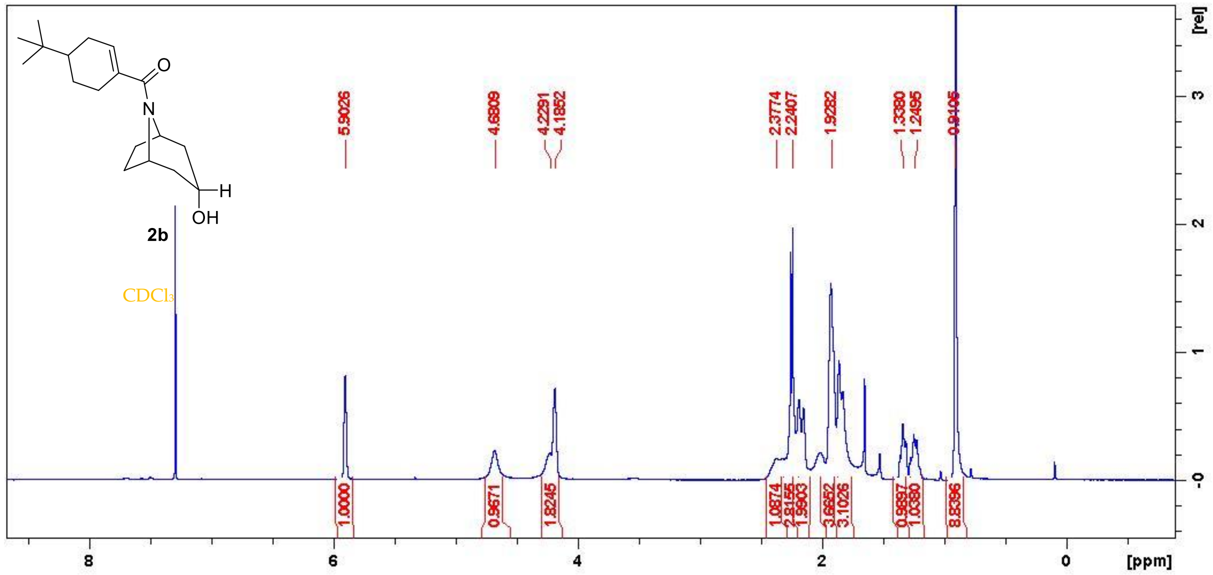

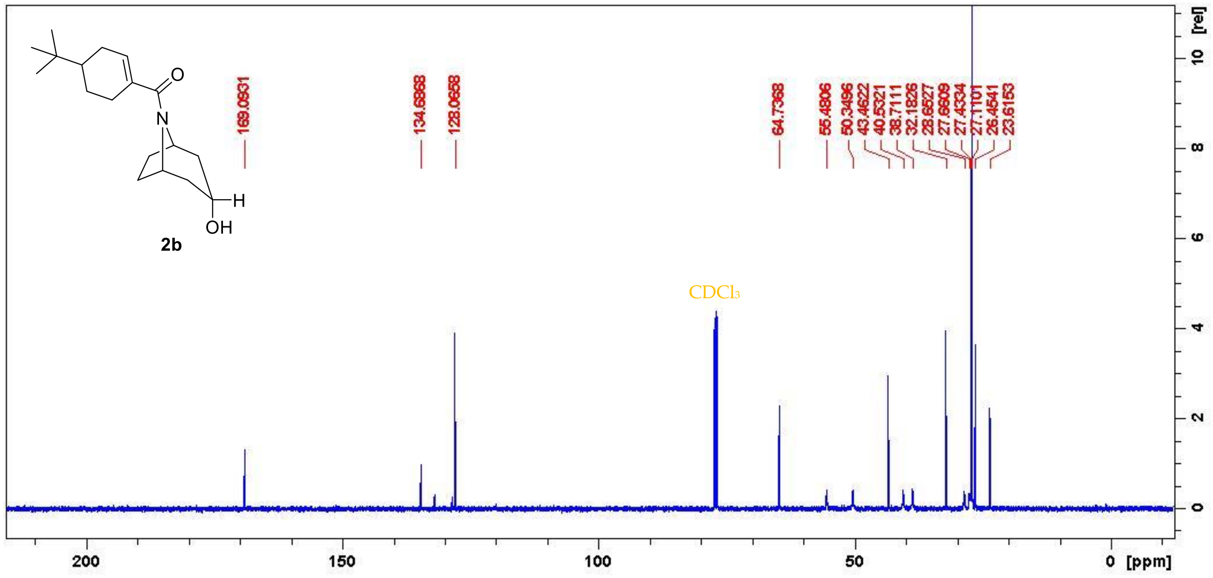

^{1}H and ^{13}C NMR spectra of **3b**

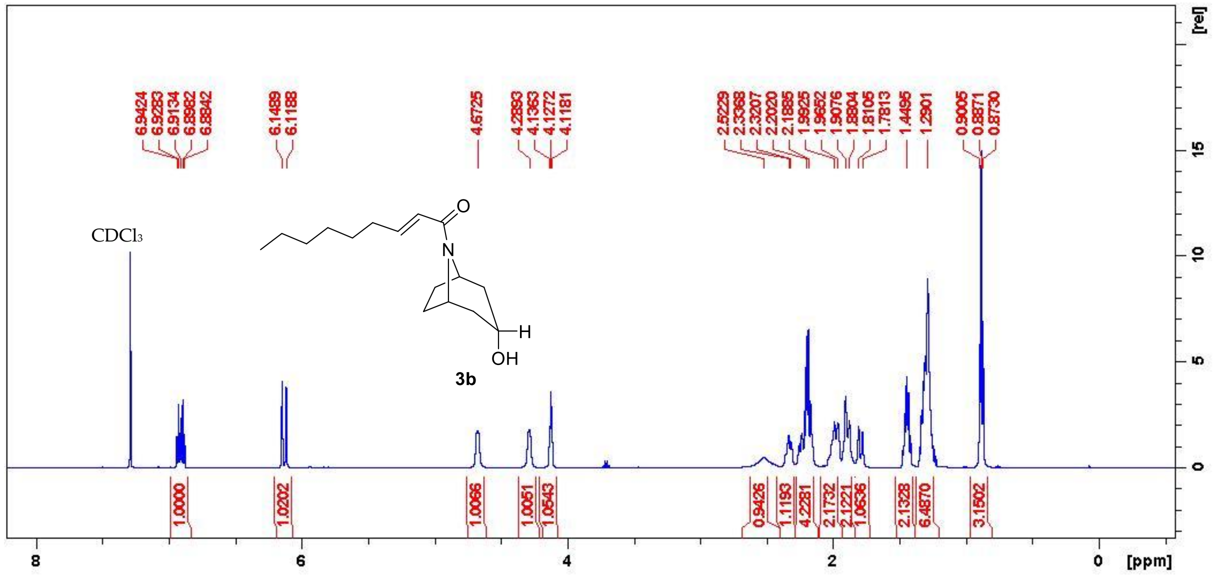

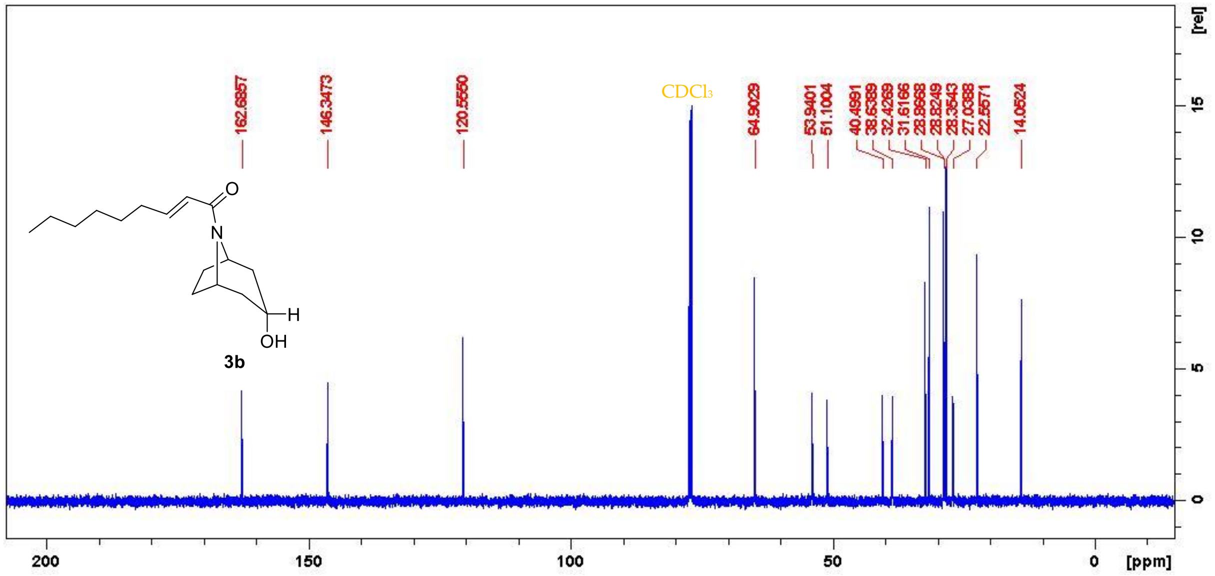

^{1}H and ^{13}C NMR spectra of **4b**

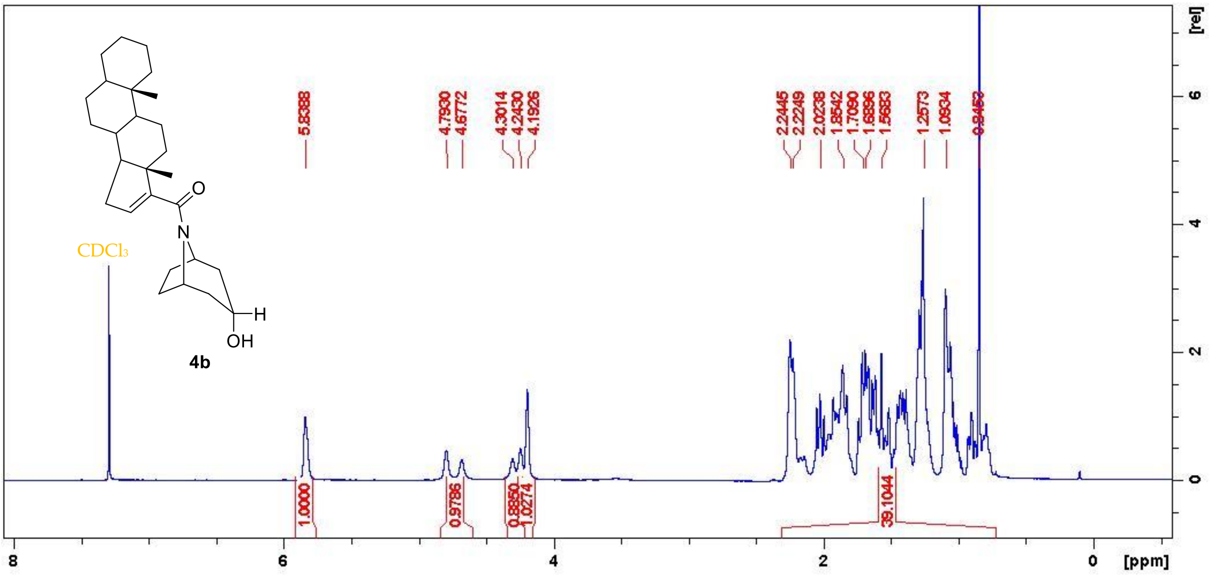

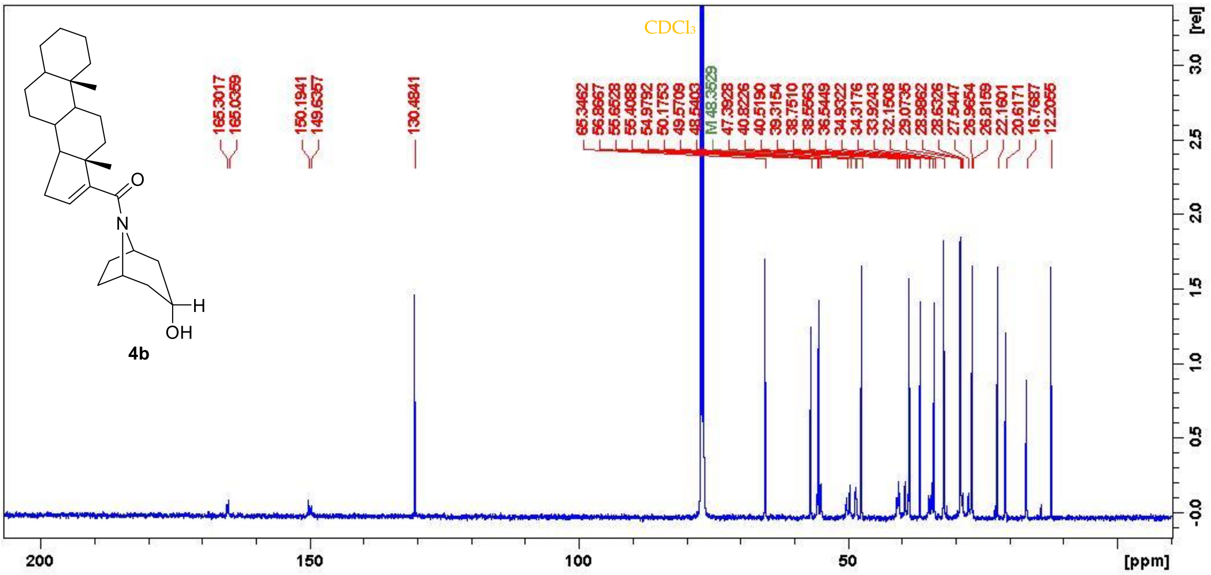

^{1}H and ^{13}C NMR spectra of **5b**

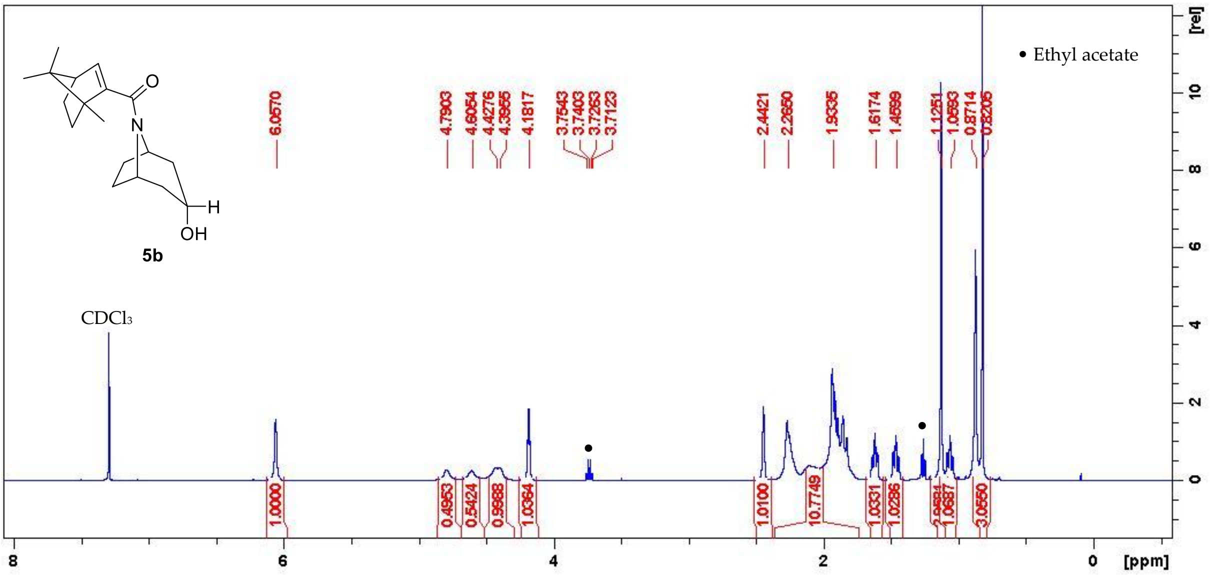

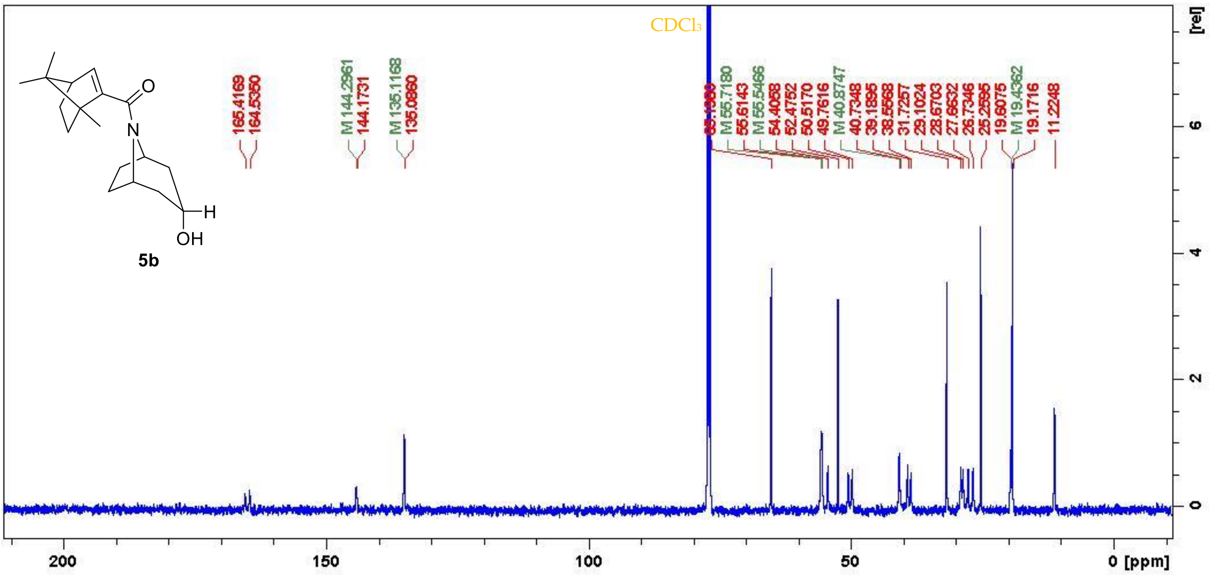

^{1}H and ^{13}C NMR spectra of **6b**

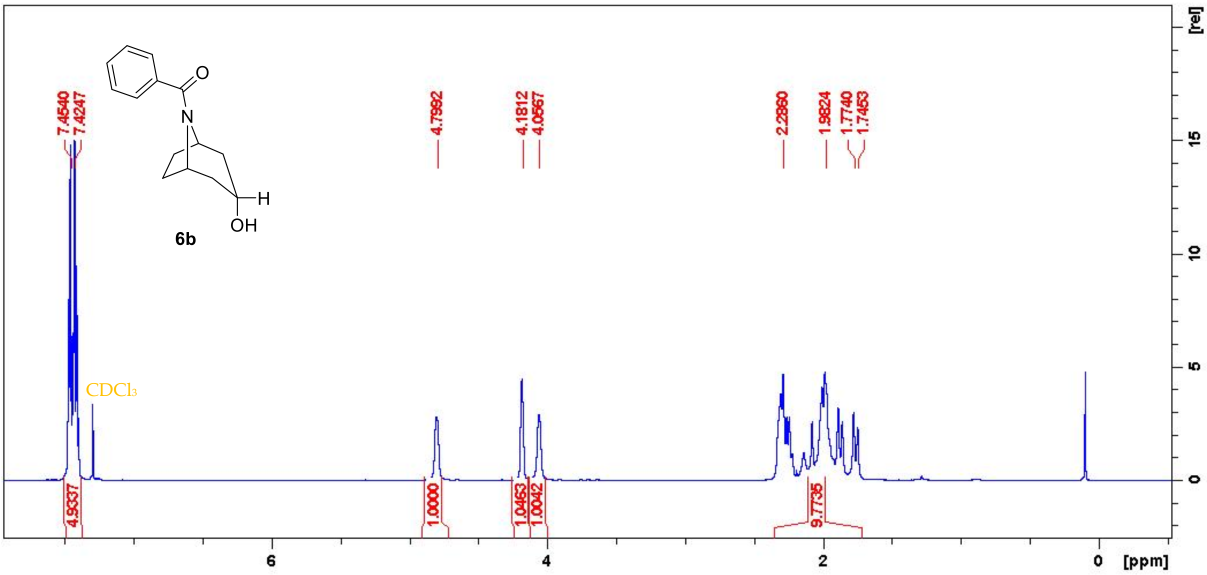

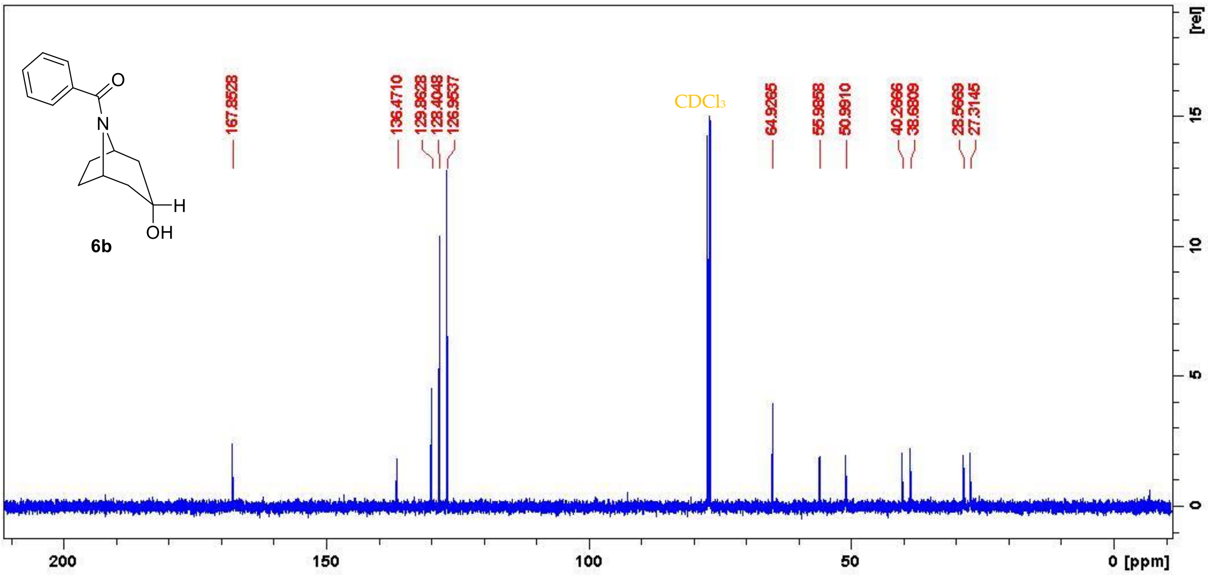

^{1}H and ^{13}C NMR spectra of **6b′**

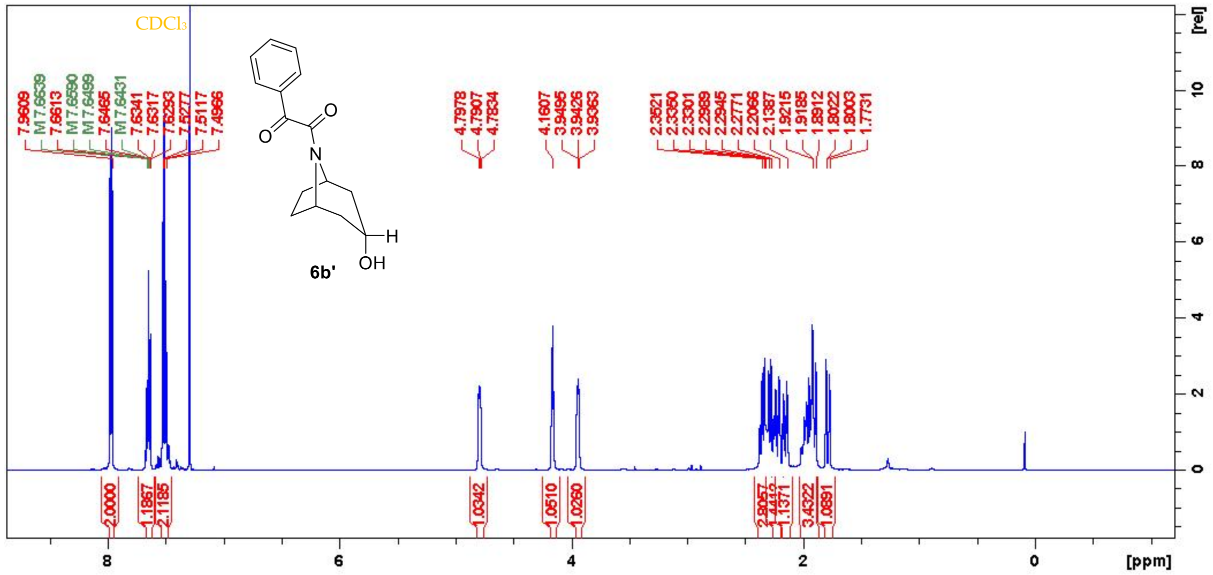

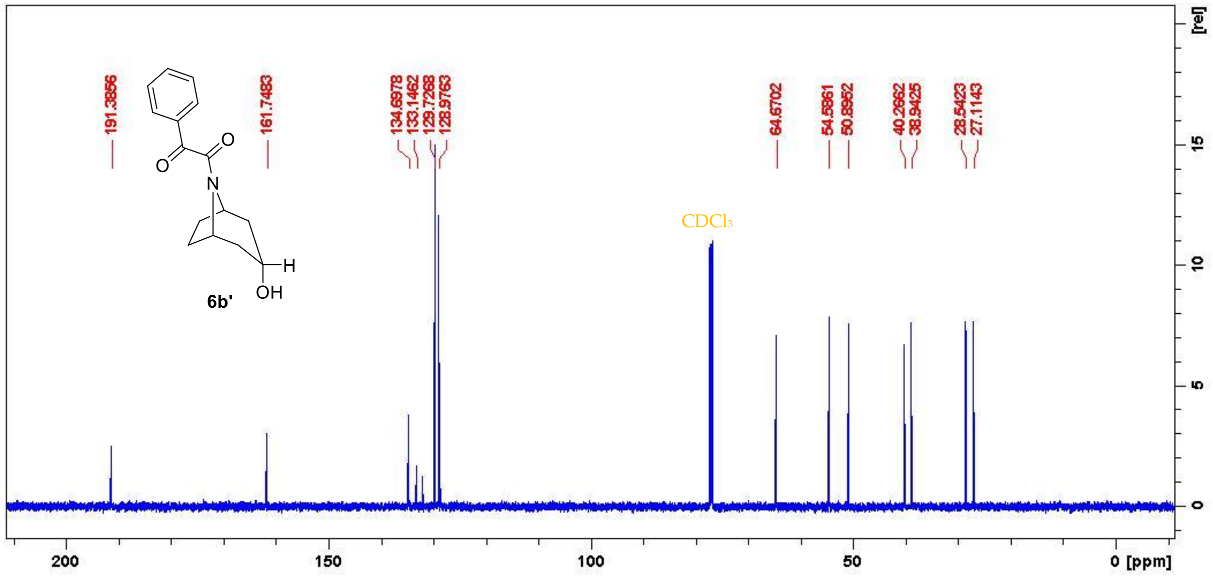

^{1}H and ^{13}C NMR spectra of **7b**

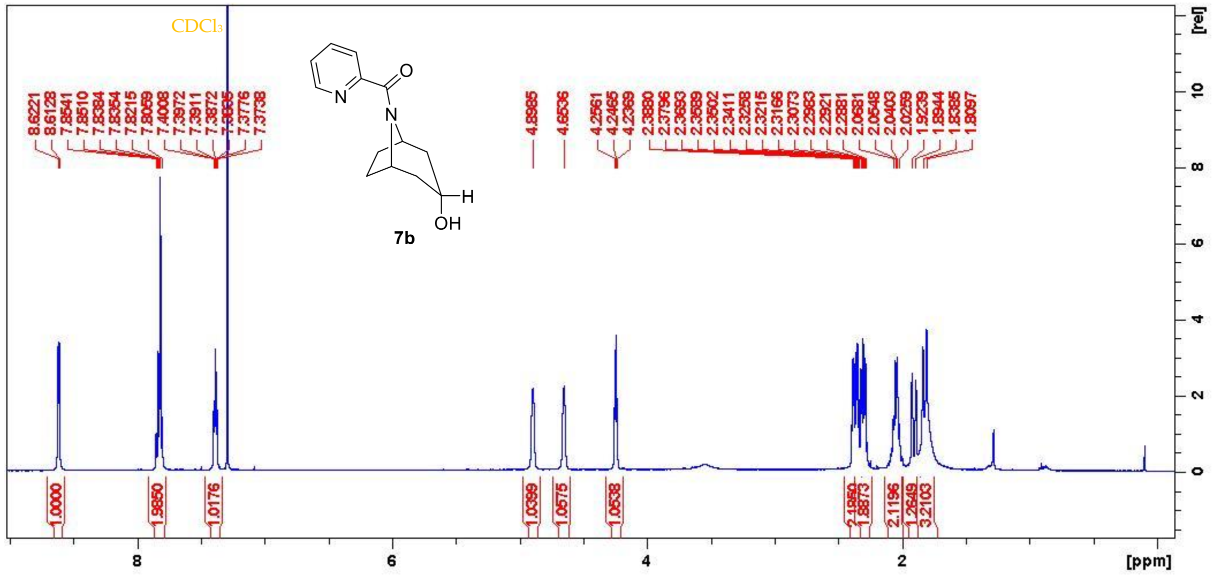

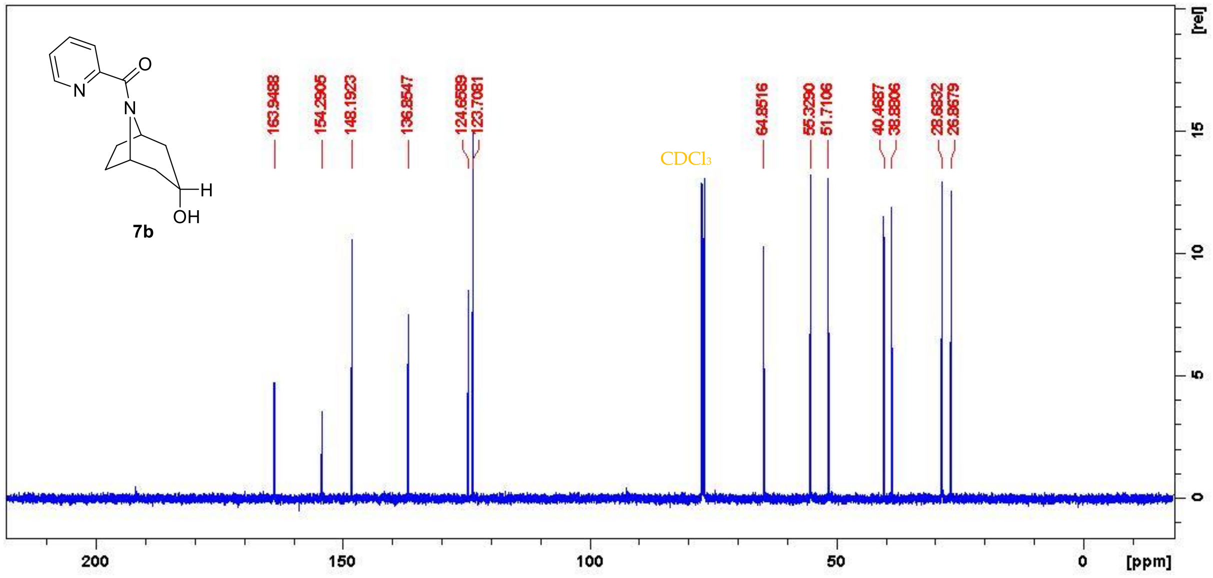

^{1}H and ^{13}C NMR spectra of **8b**

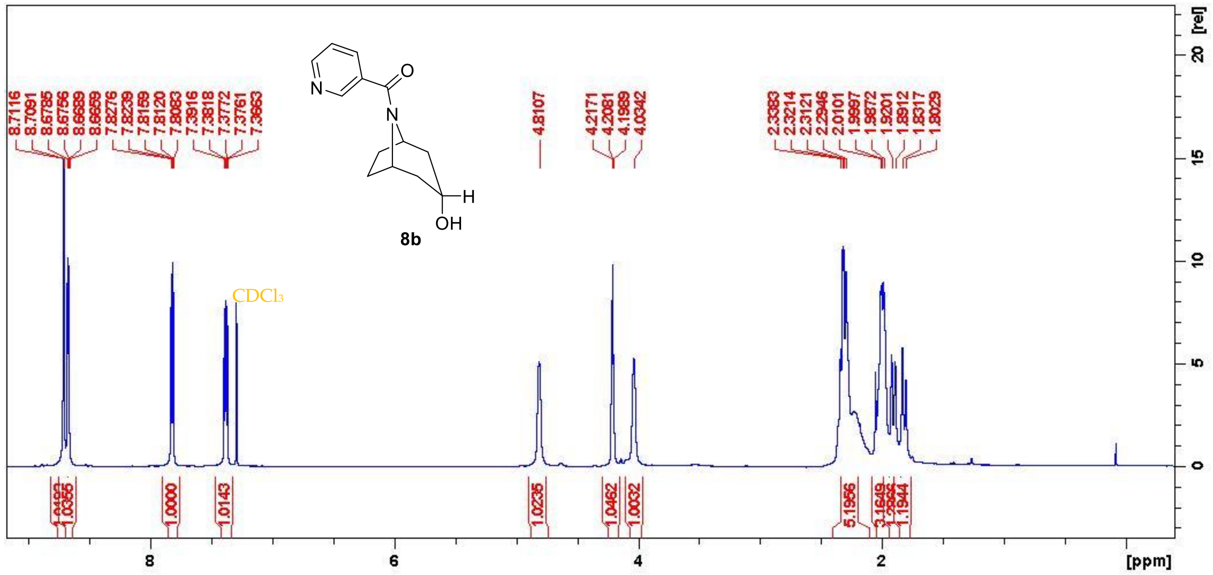

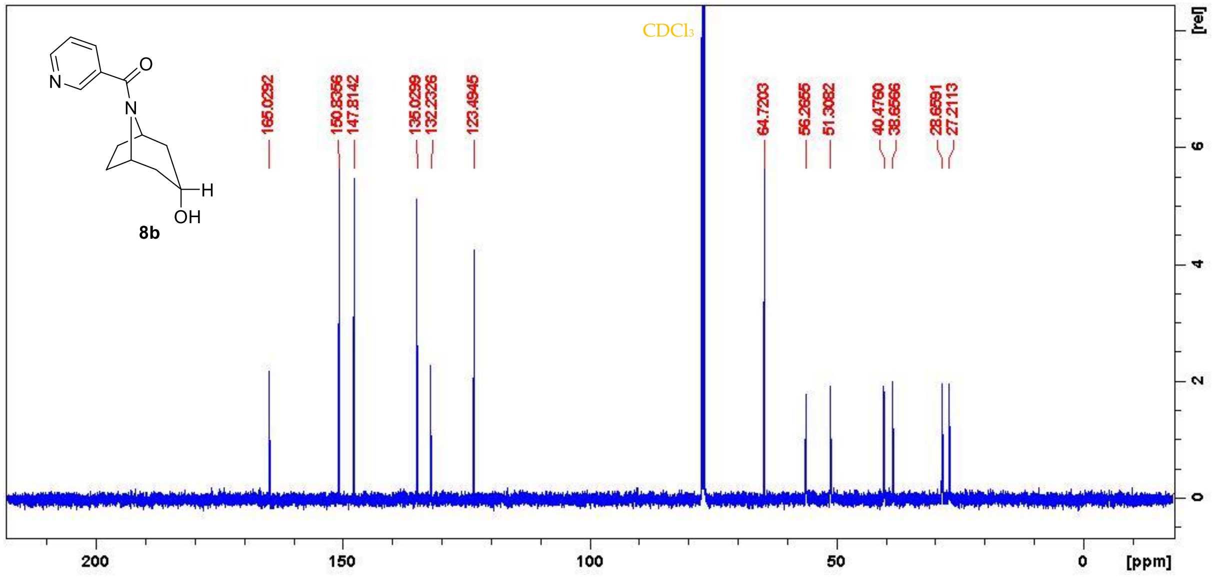

^{1}H and ^{13}C NMR spectra of **9b**

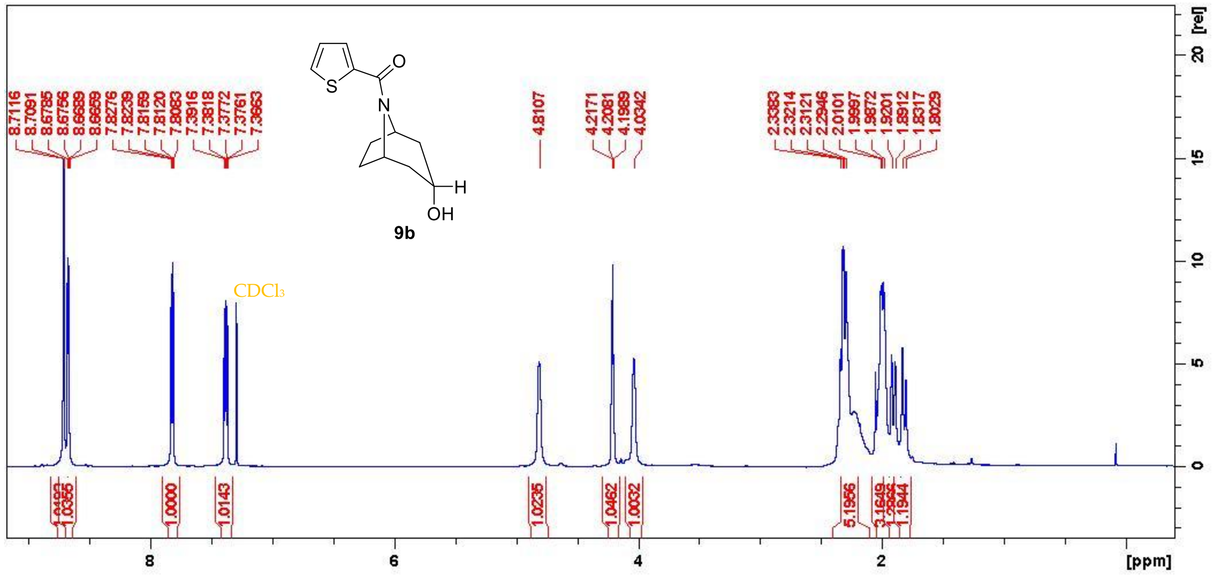

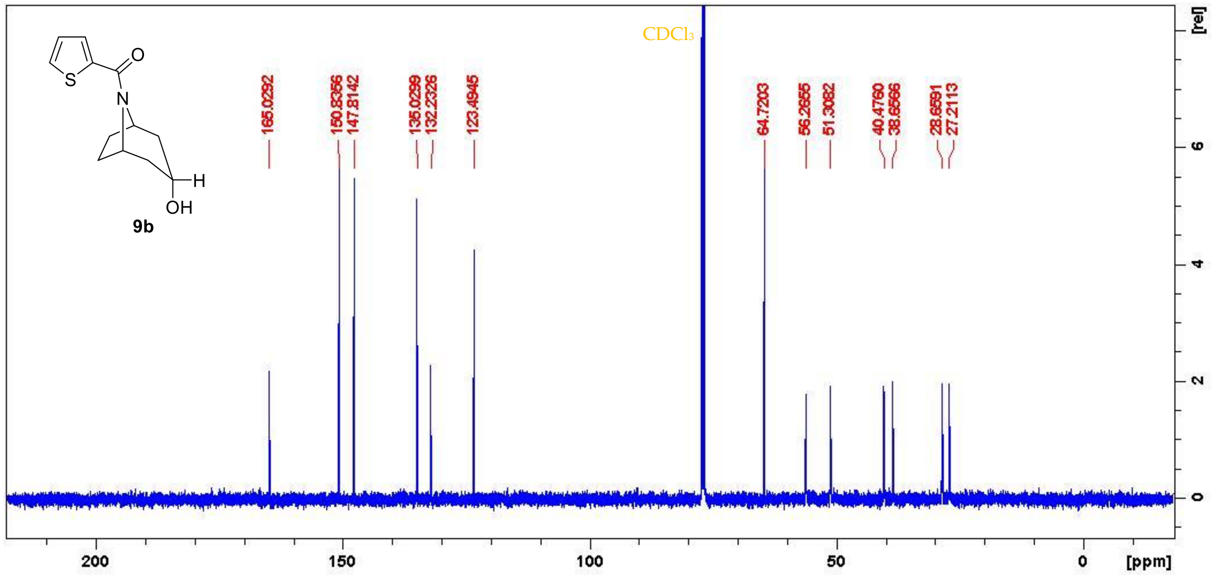

¹H and ¹³C NMR spectra of **10b**

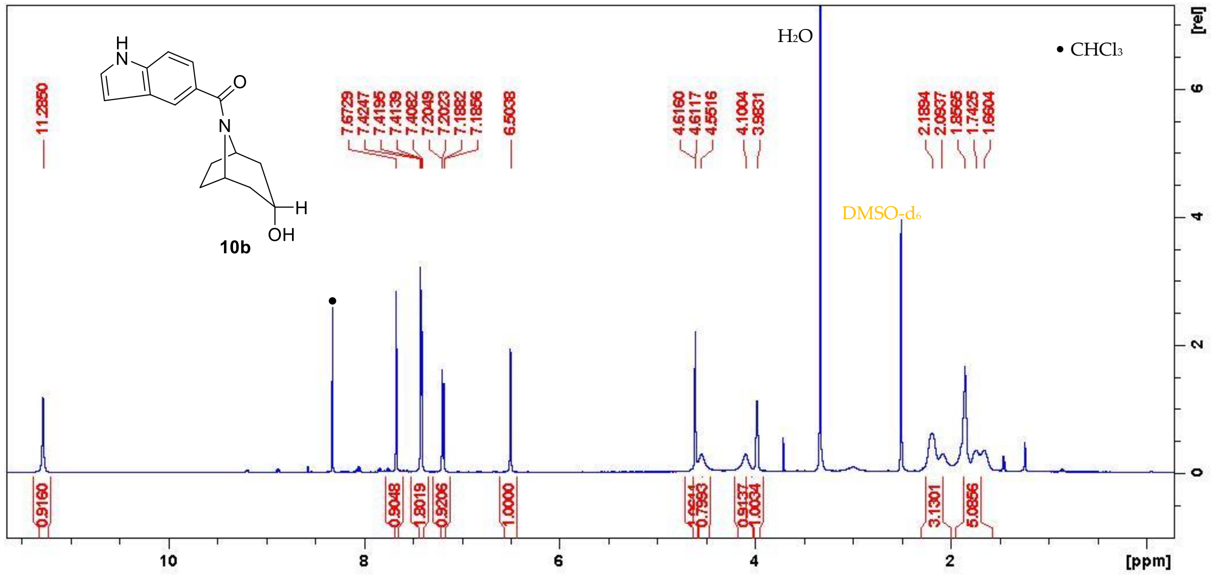

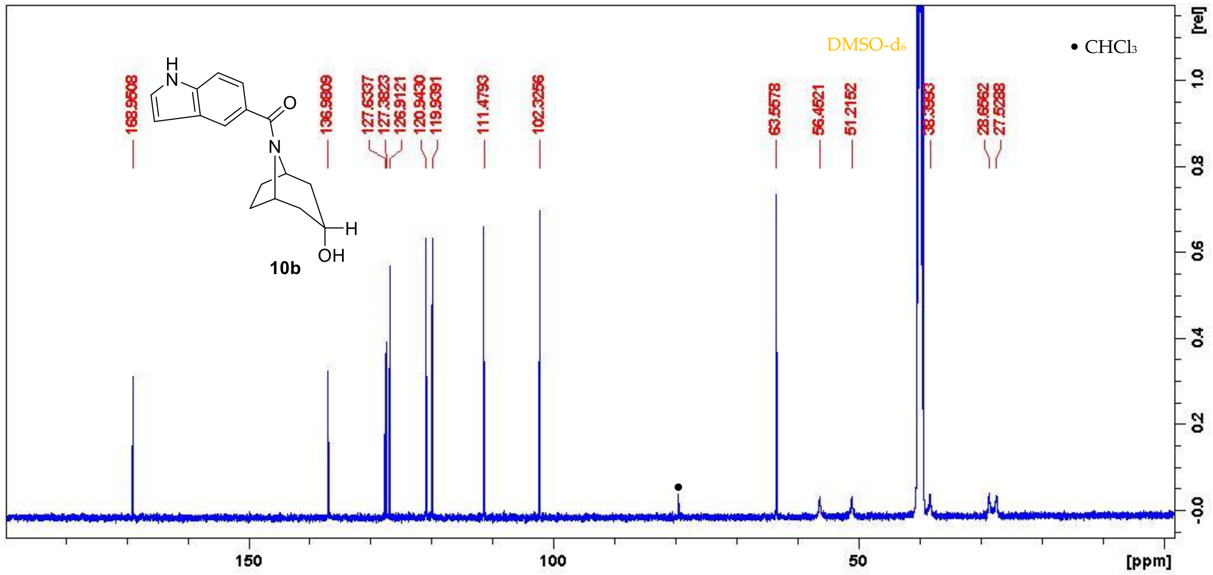

Supplement: Supplementary file 1 [file molecules-26-01813-s001.pdf]
